# Lipid Profiling following Intake of the Omega 3 Fatty Acid DHA Identifies the Peroxidized Metabolites F_4_-Neuroprostanes as the Best Predictors of Atherosclerosis Prevention

**DOI:** 10.1371/journal.pone.0089393

**Published:** 2014-02-18

**Authors:** Cécile Gladine, John W. Newman, Thierry Durand, Theresa L. Pedersen, Jean-Marie Galano, Céline Demougeot, Olivier Berdeaux, Estelle Pujos-Guillot, Andrzej Mazur, Blandine Comte

**Affiliations:** 1 UMR1019 Unité de Nutrition Humaine (UNH), INRA, CRNH Auvergne, Clermont Université, Université d’Auvergne, Clermont-Ferrand, France; 2 Obesity and Metabolism Research Unit, USDA, ARS, Western Human Nutrition Research Center, Davis, California, United States of America; 3 Department of Nutrition, University of California, Davis, California, United States of America; 4 Institut des Biomolécules Max Mousseron (IBMM), UMR CNRS 5247, Universités de Montpellier I et II, France, Montpellier, France; 5 EA 4267 Fonctions et Dysfonctions epithéliales, University of Franche-Comté, Besançon, France; 6 UMR6265 Centre des Sciences du Goût et de l’Alimentation, CNRS, Dijon, France; 7 UMR1324 Centre des Sciences du Goût et de l’Alimentation, INRA, Dijon, France; 8 UMR Centre des Sciences du Goût et de l’Alimentation, Université de Bourgogne, Dijon, France; 9 UMR 1019, Plateforme d’Exploration du Métabolisme, INRA, Clermont-Ferrand, France; Max Delbrueck Center for Molecular Medicine, Germany

## Abstract

The anti-atherogenic effects of omega 3 fatty acids, namely eicosapentaenoic (EPA) and docosahexaenoic acids (DHA) are well recognized but the impact of dietary intake on bioactive lipid mediator profiles remains unclear. Such a profiling effort may offer novel targets for future studies into the mechanism of action of omega 3 fatty acids. The present study aimed to determine the impact of DHA supplementation on the profiles of polyunsaturated fatty acids (PUFA) oxygenated metabolites and to investigate their contribution to atherosclerosis prevention. A special emphasis was given to the non-enzymatic metabolites knowing the high susceptibility of DHA to free radical-mediated peroxidation and the increased oxidative stress associated with plaque formation. Atherosclerosis prone mice (LDLR^−/−^) received increasing doses of DHA (0, 0.1, 1 or 2% of energy) during 20 weeks leading to a dose-dependent reduction of atherosclerosis (R^2^ = 0.97, p = 0.02), triglyceridemia (R^2^ = 0.97, p = 0.01) and cholesterolemia (R^2^ = 0.96, p<0.01). Targeted lipidomic analyses revealed that both the profiles of EPA and DHA and their corresponding oxygenated metabolites were substantially modulated in plasma and liver. Notably, the hepatic level of F_4_-neuroprostanes, a specific class of DHA peroxidized metabolites, was strongly correlated with the hepatic DHA level. Moreover, unbiased statistical analysis including correlation analyses, hierarchical cluster and projection to latent structure discriminate analysis revealed that the hepatic level of F_4_-neuroprostanes was the variable most negatively correlated with the plaque extent (p<0.001) and along with plasma EPA-derived diols was an important mathematical positive predictor of atherosclerosis prevention. Thus, oxygenated n-3 PUFAs, and F_4_-neuroprostanes in particular, are potential biomarkers of DHA-associated atherosclerosis prevention. While these may contribute to the anti-atherogenic effects of DHA, further *in vitro* investigations are needed to confirm such a contention and to decipher the molecular mechanisms of action.

## Introduction

Consumption of long chain omega-3 polyunsaturated fatty acids (LC n-3 PUFAs), notably eicosapentaenoic acid (EPA) and docosahexaenoic acid (DHA), has been reported to improve the prognosis of several chronic diseases related to inflammation and oxidative stress [1],[2]. Regarding cardiovascular diseases, protective effects of LC n-3 PUFAs can be in part ascribed to reduced athero-thrombotic events [3],[4],[5]. These are attributable to the modulation of specific risk factors such as reduction of platelet aggregation, decrease of plasma triglycerides (TG) and blood pressure (BP), as well as a direct regulation of systemic and local inflammation underlying plaque inception, progression and instability [3],[5]. Molecular mechanisms of action of LC n-3 PUFAs have been extensively studied but research gaps remain, particularly on the identification of the oxygenated metabolites which are increasingly considered as major effectors of the LC n-3 PUFAs. To date, research has been mainly focusing on the enzymatic oxygenated metabolites of LC n-3 PUFAs. They comprise the well-known eicosanoids which are produced from EPA and involve cyclooxygenase (producing 3-series prostaglandins and thromboxanes) and 5-lipoxygenase (producing 5-series leukotrienes). An alternative enzymatic pathway involving the 5- and 15-lipooxygenases and generating resolvins, protectins and maresins from both EPA and DHA have been more recently described [6]. Various alcohols, ketones, epoxides and diols can also be produced from LC n-3 PUFAs by the independent or coordinated action of lipoxygenases, peroxidases, alcohol dehydrogenases, cytochrome P450 epoxygenases and epoxide hydrolase [7]. The non-enzymatic pathways also referred to as the free-radical-mediated peroxidation pathway has been much less considered as a putative source of bioactive n-3 PUFAs metabolites. However, LC n-3 PUFAs, and DHA in particular, are highly prone to free-radical-mediated peroxidation [8] which produce an array of metabolites from hydroperoxide decomposition and rearrangement including hydroxyhexenal (HHE) and the isoprostanes/neuroprostanes (IsoPs/NeuroPs) [9],[10],[11]. Moreover, the free radical-mediated peroxidation of DHA is likely a major metabolic pathway during atherogenesis because of the enhanced production of free radicals in the artery wall [12]. This emphasizes a conceptual paradox between the atheroprotective properties of DHA and its susceptibility to peroxidation during atherogenesis. We thus hypothesized that non-enzymatic oxygenated metabolites generated from DHA could also play a role in atherosclerosis prevention. To broaden our understanding of metabolic changes associated with atherosclerosis progression in the presence and absence of DHA, we designed experiments to ask two specific questions: 1) What is the impact of DHA supplementation on the profiles of PUFA oxygenated metabolites? 2) Is there a relationship between the production of oxygenated metabolites and the atherosclerotic plaque progression? To address these questions, we conducted a dose-response intervention study with DHA in atherosclerosis-prone LDLR^−/−^ mice and used targeted lipidomic analyses to quantify PUFA-derived oxygenated metabolites in plasma and liver. Multivariate analysis methods including correlation analyses, hierarchical cluster and projection to latent structure discriminate analysis (PLS-DA) were finally used to investigate relationships between plaque extent and the levels of PUFA oxygenated metabolites. This integrated biological and biostatistical analysis resulted in oxygenated PUFA metabolite profiling and the identification of potent predictive variables of atherosclerosis.

## Materials and Methods

### Ethics Statement

This study was carried out in strict accordance with the Institutional Ethics Committee of the INRA. The protocol was approved by the Committee on the Ethics of Animal Experiments of the Auvergne Region (Permit Number: CE-2910). All efforts were made to minimize suffering during the protocol and before the final experiment.

### Mice, Diets and Study Outline

LDLR^−/−^ mice (n = 120) were purchased from Jackson Laboratories (Charles River Laboratories, L’Arbresle, France), housed 10 per cage, in a temperature controlled environment (22±0.8°C) with a 12-h light–dark cycle, and allowed free access to food and water. At 8 weeks of age, mice were given a diet enriched in animal fat (10% of lard) and low in cholesterol (0.045%, Sigma-Aldrich C75209), and were randomized into four different groups on the basis of body weight. In parallel to the diet, mice received by daily oral gavages (50 µL, 5 days/week) of either oleic acid rich sunflower oil (Lesieur, Asnières-sur-Seine, France; Control group) or a mixture of oleic acid rich sunflower oil and DHA rich tuna oil (OMEGAVIE DHA 90 TG, Polaris Nutritional Lipids, France containing 90% of DHA as TG). This oil mixture provided 0.1% (or 1.77 mg/d/mouse), 1% (or 17.7 mg/d/mouse) or 2% (or 35.5 mg/d/mouse) of energy as DHA (DHA1, DHA2 and DHA3 groups respectively). These 3 doses of DHA were chosen first to be close to nutritional conditions (recommended intake for human: 0.1% of energy [13]) and secondly to levels reported in the literature [14]. Moreover, to avoid oxidation of oil mixtures, preparations were performed under nitrogen, aliquoted into 3 mL cryotubes, corresponding to daily doses of gavage and stored in the dark at −80°C until use. After 20 weeks of feeding, mice were anaesthetized (40 mg pentobarbital/kg body weight), blood was drawn into sodium EDTA (200 mM) and plasma was rapidly collected and stored at −80°C until further utilization. After rapid washing with sterilized PBS, liver and aorta samples were immediately frozen into liquid nitrogen and stored at −80°C.

### Blood Pressure and Heart rate

Systolic blood (sBP) and diastolic blood pressures (dBP) as well as heart rate were measured in conscious mice using the indirect tail-cuff method (BP 2000, Visitech System, Apex, North Carolina, USA). Measurements were performed at the beginning and at the end of the experimental period.

### Quantification of Atherosclerotic Lesions

Atherosclerotic lesions were assessed by measuring lipid deposit in the aortic sinus as previously described [15]. Briefly, four sections of 10 µm thickness were harvested/slide and 28 slides/mouse were prepared and stained with Oil red O (Merck, Darmstadt, Germany) and counterstained with hematoxylin (Diapath, Martinengo, Italy). Each section was evaluated for Oil red O staining area under microscope. Image analysis was carried out using the ImageJ free software (http://rsb.info.nih.gov/ij/) to quantify the cross-section surface areas of the lesions and the cross-section surface area of the aorta. The lesion area was calculated by dividing the surface of the lesion by the surface of the vessel and expressed as a percentage. This approach allows correcting for errors caused by oblique sections that may lead to overestimation of the surface area occupied by a lesion.

### Plasma and Liver Lipids

Plasma total cholesterol and TG concentrations were determined as previously described [16]. Liver samples were homogenized in NaCl (9 g.l^−1^) with a Polytron homogeniser PT-MR2100 (Kinermatica AG, Littau/Luzern, Switzerland) and lipids were extracted by chloroform–methanol (2∶1, v/v) under overnight agitation. The chloroform phase was recovered after centrifugation and evaporated under dry air. TAGs from the lipid residue were saponified with 0.5 M KOH–ethanol at 70°C for 30 min followed by the addition of 0.15 M MgSO_4_ to neutralize the mixture. After centrifugation (2,000×*g*; 5 min), glycerol from TAG in the supernatant was quantified by an enzymatic assay (TG PAP 150 kits, BioMerieux, Marcy-l’Etoile, France). Cholesterol in the lipid residue was dissolved with isopropanol and measured using enzymatic commercial kit (Cholesterol RTUTM, BioMerieux, Marcy-l’Etoile, France).

### Plasma Fatty Acids and Oxylipins

Esterified plasma lipids were quantified by mass spectrometry after total lipid extraction in the presence of fatty acid, cholesterylester, triglyceride, and phospholipid surrogates. Extracts were split for the independent quantification of esterified fatty acids and alkali stable oxylipins. Esterified fatty acids were transformed to fatty acid methyl esters and quantified by GC-MS. Fractions for oxylipin analyses were enriched with isotopically labeled oxylipin fatty acids, subjected to base hydrolysis, and the resulting free oxylipins were isolated by solid phase extraction (SPE) and quantified by LC-MS/MS.

Specifically, plasma aliquots (100 µL) were enriched with 5 µL 0.2 mg/ml butylated hydroxytoluene/EDTA in 1∶1 methanol:water, and a suite of extraction surrogates, which included deuterated-tri-palmitoyl glycerol (d31-16∶0-TG; CDN Isotopes, Pointe-Claire, Quebec, Canada), deuterated distearoylphosphotidylcholine (d35-18∶0-PC; Avanti Polar Lipids, Alabaster, Alabama), dodeca-(9E)-enoyl cholesterylesters (22∶1n9-CE; NuChek Prep, Elysian MN) and dodecatrienoic acid (22∶3n3; NuChek Prep). Lipids were then extracted with 10∶8: 11 cylcohexane: 2-propanol:ammonium acetate as described by Smedes [17]. Briefly, enriched samples were mixed with cyclopropane/2-propanol, phases were split with ammonium acetate, the organic phase was isolated and the aqueous phase was re-extracted with cyclohexane. The combined organic total lipid extract was reduced to dryness and reconstituted in 100 µL of 1∶1 methanol/toluene.

A sub-aliquot of the total lipid extract was used to quantify plasma fatty acids as methyl esters by gas chromatography-mass spectrometry (GC-MS). Extract aliquots (10 µL) were spiked with 15∶1n5 free acid to track methylation efficiency and brought to a final volume of 200 µL with 90∶10 methanol/toluene (v/v) and incubated at 60°C for 1hr with 100 µL 0.5 M sodium methoxide, followed by 1 hr with 100 µL 0.5 M methanolic HCl. Solutions were then neutralized with 400 µL 0.25M KHCO_3_/0.5M K_2_CO_3_ and fatty acid methyl esters (FAMEs) were extracted into 400 µL hexane, and extracts were washed with 400 µL saturated saline. A 60 µL extract aliquot was enriched with 10 µL of 44 µM tricosanoate methyl ester (23∶0; NuChek Prep). A 1 µL injection was analyzed by GC-MS on an Agilent 6890/5973N with electron impact ionization and in simultaneous selected ion monitoring/full scan mode. Analytes were separated on a 30 m×0.25 mm×0.25 µm DB-225 ms as previously described [18].

Total alkali stable plasma oxylipins were quantified in total lipid extracts by UPLC-MS/MS after the isolation of saponified oxylipins by SPE. Total lipid extract sub-aliquots (40 µL 1∶1 methanol/toluene) were enriched with a suite of 12 deuterated prostanoids, eicosanoids, and octadecanoid free acid surrogates (Table S1 in File S1), in 5 µL methanol. Enriched aliquots were incubated at 60°C for 1 hr with 100 µL 0.5 M sodium methoxide, followed by 1 hr after the addition of 100 µL of water to hydrolyze methyl esters. Hydrolysis of methyl esters in 1∶1 MeOH/water has been previously shown to efficiently liberate alkali stable methyl esters [19],[20],[21] and trans-esterification followed by hydrolysis has been found to increase yields for lipids with low water solubility (e.g. CE and TG, data not shown). Oasis HLB SPE columns (10 mg×1cc; Waters Corporation, Milford, MA, USA) were washed with 1 mL ethyl acetate, 2 mL MeOH, and conditioned with 2 mL 5% MeOH/0.1% acetic acid SPE prior to sample extraction. Hydrolyzed samples were up-diluted with 0.5 mL 5% MeOH/0.1% acetic acid SPE, neutralized with 10 µL 20% glacial acetic acid, and transferred to SPE reservoirs along with a second 0.5 mL 5% MeOH/0.1% acetic acid rinsate, for a 16% final organic content by volume. Samples were allowed to extract by gravity, sorbent phase was washed with 1 mL of 20% MeOH/0.1% acetic acid and air dried for 30 min at −25 psi. Columns were then wetted with 0.5 mL MeOH/1% acetic acid and residues eluted with 1.5 mL of ethyl acetate into 2 mL vials containing a solution of 6 µL 30% glycerol in methanol. Residues were brought to glycerol residue under vacuum and reconstituted with 50 µL MeOH containing 100 nM each of 1-cyclohexylurea-3-dodecanoic acid (Sigma-Aldrich, St. Louis, MO) and 1-phenylurea-3-hexanoic acid, as internal standards. The sample extract was filtered by centrifugation for 3 min at 8°C using 0.1 µm Amicon Ultrafree-135 MC Durapore PVDF filters (Millipore, Billerica, MA). Five µL of filtered extract was analyzed, and analytes of interest were separated by reverse phase ultra-performance liquid chromatograph with a 1.7 µm Acquity BEH column (Waters, Milford, MA) using a 16 min gradient (Solvent A = 0.1% acetic acid; Solvent B = 90∶10 v/v acetonitrile/isopropanol; see Table S2 in File S1 for details). It should be noted, as showed in Table S1 in File S1, that the apparent surrogate recoveries were lower than typically observed with these methods, particularly for the epoxy fatty acids, which may reduce the accuracy in the final data. However, as replicate precision was high, the reported data is considered valid for the determination of the treatment effect investigated in the present study.

Oxylipins were detected on an API 4000 QTrap (AB Sciex, Framingham, MA, USA) by multiple reaction monitoring (MRM) after negative mode electrospray ionization, and quantified against 6pt calibration curves using internal standard methodologies. Analyte retention times, mass transitions, mass spectral parameters and surrogate/internal standard associations, and estimated detection limits are presented in Table S3 in File S1. Our analytical criteria for reporting are as follows: data >3∶1 signal to noise; the relative contribution of background from method blanks is <25% of signal; values are within the calibrated range.

It should be noted that alkaline conditions destroy the β-hydroxy-keto prostanoids (e.g. PGEs, PGDs) and thromboxanes, as well as cystienyl leukotrienes and ketones (Tables S8, S9, and S10 in File S1) but not the β-hydroxy-alcohol PGFs [22] or other oxylipin classes analyzed here (see Table S1 in File S1). While plasma deuterated PGF2a showed good recoveries and F2-isoprostanes were detected in ∼70% of samples, concentrations were uniformly low, <4× the average blank values, and are not reported here. To provide readers an estimate of the lowest detectable concentration, the lowest detectable calibrated concentration has been transformed to a plasma equivalent concentration for each analyte and included in Table S8 in File S1. Finally, whereas artificial formation of oxylipins during saponification cannot be completely excluded, the use of EDTA to chelate free metals which can catalyze auto-oxidative degradation of lipids and the use of butylated hydroxyl toluene as an anti-oxidant should have limited this process. Moreover, as shown in Figure S6 in File S1, the auto-oxidative markers F2-isoprostanes and 9-HETE levels were low and did not co-vary. Finally, while some oxylipins were strongly covariant with 9-HETE levels in the samples, others were not. Importantly, while 9-HETE was correlated with 11-HETE and 15-HETE concentrations, 12-HETE and plasma epoxide concentrations were not (Figure S7 in File S1). Taken together these factors suggest that oxylipins were not formed during sample processing.

### Liver Fatty Acids

Total lipids were extracted from liver samples following a the choloroform:methanol procedures of Folch *et al.* as previously described [23], and fatty acid methyl esters were prepared for analysis by gas chromatography-flame ionization detection. Briefly, after drying with anhydrous sodium sulfate, the organic extract was evaporated under nitrogen and total lipids dissolved with toluene/methanol (1/2, v/v). FAMEs were obtained after trans-esterification followed by acid trans-esterification. GC analysis of FAMEs was performed using a GC Trace (Thermo Fischer Scientific, Courtaboeuf, France), equipped with a fused silica CP-Sil 88 capillary column (100% cyanopropyl-polysiloxane, 100 m×0.25 mm×0.20 µm; Varian S.A, Les Ulis, France). The identities of sample methyl esters were determined by comparing their relative retention times with those of external FAME standards (Supelco 37 Component Fatty Acid Methyl Esters Mix and Menhaden Oil; Sigma Aldrich, St Quentin Fallavier, France). Other standard FAME mixtures were obtained from Nu-Chek-Prep (Elysian, MN, USA).

### Liver Hydroxyalkenals

Hepatic levels of the hydroxyalkenals thioether protein adducts (hydroxynonenal or HNE-P and hydroxyhexenal or HHE-P from n-6 and n-3 PUFA respectively) were measured by GC-MS/MS as previously described [23]. Briefly, 100 mg of liver tissue were processed and immediately treated with NaB^2^H_4_ to reduce aldehydes into their chemically stable ^2^H labeled alcohol derivatives. Then, proteins were precipitated and resuspended into 8 mM guanidine buffer. The mixture was spiked with the labeled internal standard ([^2^H_11_] 4-HNE, 0.25 nmol) and incubated with Raney nickel (overnight, 55°C) to cleave the thioether linkages and reduce the C–C bonds. The free saturated derivatives were then extracted and derivatized with N-methyl-N-(*t*-butyldimethylsylyl)-trifluoroacetamide (2 hr, 80°C). Samples were analyzed by GC-MS/MS on a Agilent 6890N GC (Agilent Technologies, Palo Alto, CA, USA) equipped the a Quattro Micro tandem mass spectrometer (Waters Corporation, Manchester, UK), as previously described. The system was run in positive mode with MRM. The collision energies were adjusted to optimize the signal for the most abundant product (daughter) ions: *m*/*z* 390>258 for [^2^H]DHN (dihydroxynonene as reduced HNE), *m*/*z* 348>216 for [^2^H]DHH (dihydroxyhexene as reduced HHE), and *m*/*z* 400>268 for the internal standard [^2^H_11_]DHN using argon as collision gas. Quantification was performed using calibration curves with external standards. Quantities of HNE-P and HHE-P adducts reported in this study represent averages of duplicate sample injections.

### Liver F_4_-neuroprostanes

Sample preparation was adapted from Musiek *et al.* (2004) [24]. Briefly samples of liver tissue (10 mg) were homogenized in 2.5 ml of ice-cold chloroform/methanol (2/1, v/v) containing butylated hydroxyl toluene (0.004%) and 50 µL of the labeled internal standard ([^2^H_4_]4-F_4t_, 40 ng/mL). Esterified F_4_-NeuroPs were isolated and hydrolyzed by adding 2 ml of 1 M aqueous potassium hydroxide. The samples were acidified to pH 3 with 1 mL of 1 M HCl and diluted with 2 mL of 0.1 M formate buffer (pH 3.0). F_4_-NeuroPs were then purified by solid phase extraction using an Oasis HLB extraction cartridge (Waters Corp) as previously described [25]. Briefly, the cartridge was preconditioned with methanol and 0.1 M formate buffer (pH 3.0), and washed with acetonitrile:water (15∶85, v:v) followed by heptane. F_4_-NeuroPs were eluted by with 2 mL of hexane:ethyl acetate:propan-2-ol (30∶65: 5, v:v:v). Purified F_4_-NeuroPs were converted to pentafluorobenzyl ester/TMS ether derivatives before GC/MS analysis using the Agilent/Waters GC-MS/MS described above, operating in negative chemical ionization mode using isobutane as reagent gas. Injections (2 µL) were performed at 250°C in splitless mode. The carrier gas was high-purity helium at a constant flow rate of 1 mL/min. Chromatographic separation was performed using an 50 m×0.32 mm i.d, 0.25 µm OV-1701 (Ohio Valley Specialty Co; Marietta, OH, USA) capillary column under the following conditions: 90°C for 1 min, increased by 40°C/min until 290°C. The final temperature was held at 290°C for 6 min. MS parameters were optimized using standard solutions. Maximum sensitivity was obtained for an ion source temperature set at 120°C, an electron energy at 90 eV and an emission current at 300 µA.

The data were acquired using MRM. The collision energies of 15 eV yielded optimal product ions intensity from the fragmentation of the carboxylate anions: *m*/*z* 593>341 for F_4_-NeuroPs, and *m*/*z* 573>303 for the internal standard [^2^H_4_]4-F_4t_-NeuroP using argon as collision gas. Quantification was performed using calibration curves with external standards. The internal standard [^2^H_4_]4-F_4t_-NeuroP was synthesized according to a recent published procedure [26],[27].

### Statistical Analyses

All data are presented as means ± SEM. Data following a Gaussian distribution (test of Kolmogorov & Smirnov) and having no significant difference between SD (Bartlett’s test) were analyzed by one-way ANOVA followed by a Tukey-Kramer post-hoc analysis using GraphPad InStat version 3.06 (GraphPad Software, San Diego California USA, www.graphpad.com). The other data were analysed using a non-parametric test (Kruskal Wallis’s test). Differences were considered significant at p<0.05.

The cumulative results were also subjected to a suite of multivariate analyses including hierarchical cluster analysis and PLS-DA. Data treatment prior to multivariate analysis included missing value imputation, and normality transformation, and autoscaled i.e. each variable was mean centred and scaled to unit variance [28]. Where variable data sets were greater than 90% complete, missing values were computationally imputed using a probabilistic principal components analysis [29],[30]. Data was transformed to normality using the procedures of Box and Cox [31]. Differences in means were determined using 2-tailed *t*-tests or Mann-Whitney U-tests if normality was not achieved. Data transformations, multivariate analyses and non-parametric statistics were performed using the Excel Add-In imDEV [32], which provides a graphical user interface to the R Statistical Computing Environment. Hierarchical clusters were based on a Minkowski distance matrix agglomerated using Ward’s minimum variance method. PLS-DA was performed using the SIMPLS algorithm with leave one out cross-validation, and predictive models were iteratively compared (n = 100) to those built with data randomly assigned to discriminate class data. Predictive models were constructed to either classify animals based on relative dietary n-3 content [discriminate classes: 0 = Control; 1 = DHA1; 10 = DHA2; 20 = DHA3] or atherosclerotic plaque area modeled as a continuous variable. Model variable selection with correlation, ANOVA, entropy minimization, and chi square filtering were compared between 10 independent training/test set selections. Correlation based filtering provided the model set with the lowest variance in the root mean squared error of prediction.

## Results

### Effects of DHA Supplementation on Cardiovascular Risk Parameters and Atherosclerosis

**Figure 1:**
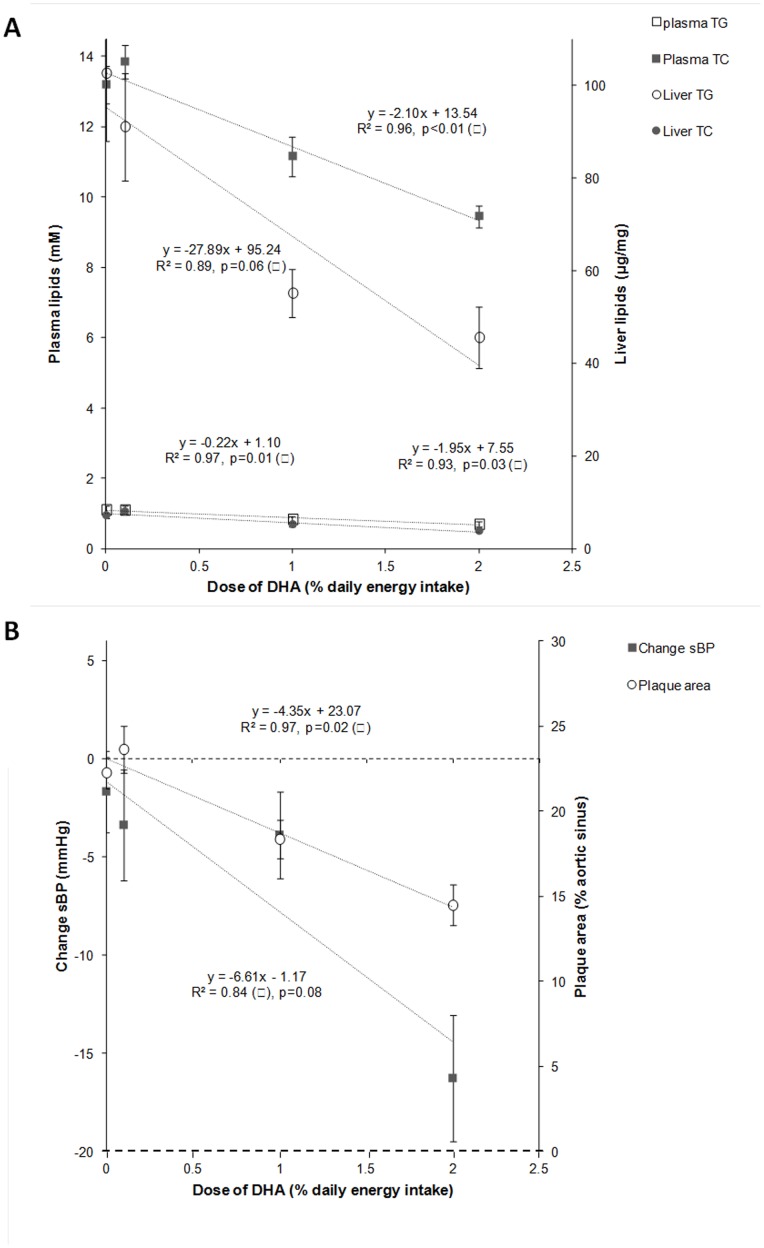
Correlations between the doses of DHA and the cardiovascular parameters (i.e. plasma TG and TC, liver TG and TC, sBP, and plaque area). LDLR^−/−^ mice were given by daily oral gavages (20 weeks) either oleic acid rich sunflower oil (Control group) or a mixture of oleic acid rich sunflower oil and DHA rich tuna oil providing 0.1%, 1% or 2% of energy as DHA (DHA1, DHA2, and DHA3 groups respectively).

**Figure 2:**
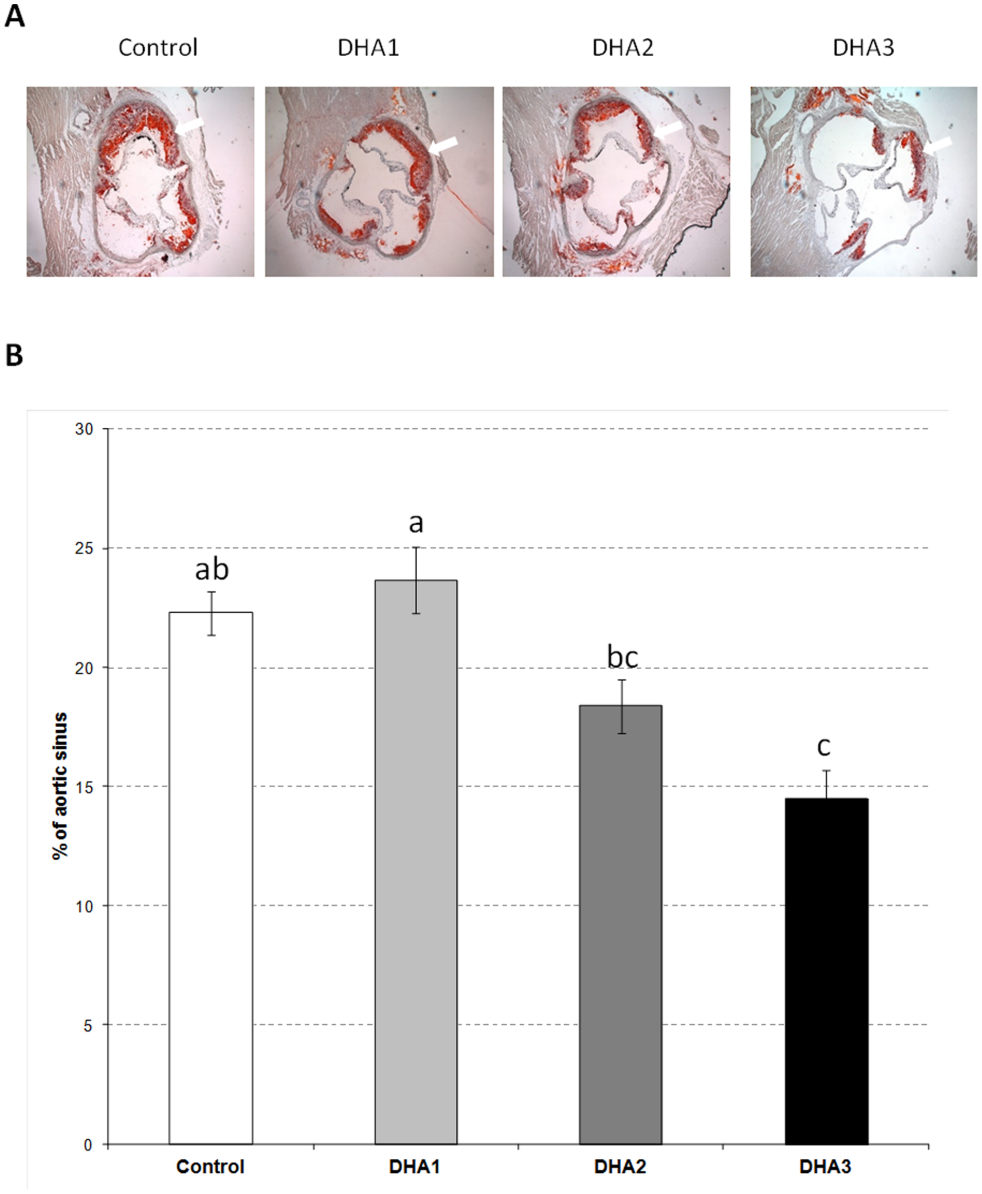
Quantification of atherosclerotic lesions. (A) Sections of aortic sinus (at 500 µm from the cusps) stained with oil red O. The arrows indicate lipid deposit. (B) Atherosclerotic lesion severity is expressed as percent changes of lesion area in total cross-sectional area. Data represent means ± SEM (n = 20/group). ^a,b,c^ Mean values with unlike letters were significantly different (p<0.05).

The dose-response effects of DHA supplementation were evaluated on several cardiovascular risk factors (i.e. plasma and hepatic lipids, sBP) and atherosclerosis as shown in Figure 1. The doses of DHA given to the LDLR^−/−^ mice (0, 0.1, 1 and 2% of energy as DHA) were negatively correlated with plasma TG and TC (R^2^ = 0.97, p = 0.01 and R^2^ = 0.96, p<0.01 respectively) as well as with liver contents of TG and TC (R^2^ = 0.89, p = 0.06 and R^2^ = 0.93, p = 0.03) (Figure 1A). There was also a strong negative correlation with atherosclerotic plaque area (R^2^ = 0.97, p = 0.02; Figure 1B). With sBP, the correlation did not reach significance (R^2^ = 0.84, p = 0.08; Figure 1B). The effects of DHA on cardiovascular risk parameters and atherosclerosis were especially strong in the DHA3 group (Table S4 in File S1) in which plasma levels of TG and TC were reduced (p<0.05) by 37 and 28% respectively, while liver concentrations of TG and TC were decreased (p<0.05) by 56 and 47% respectively. Concerning atherosclerotic plaque extent (Figure 2) and sBP (Table S5 in File S1), the reductions induced by DHA supplementation were also especially pronounced in the DHA3 group (−35 and −16%, p<0.05 respectively).

### Impact of DHA Supplementation on the Profiles of Fatty Acids in Plasma and Liver

**Figure 3:**
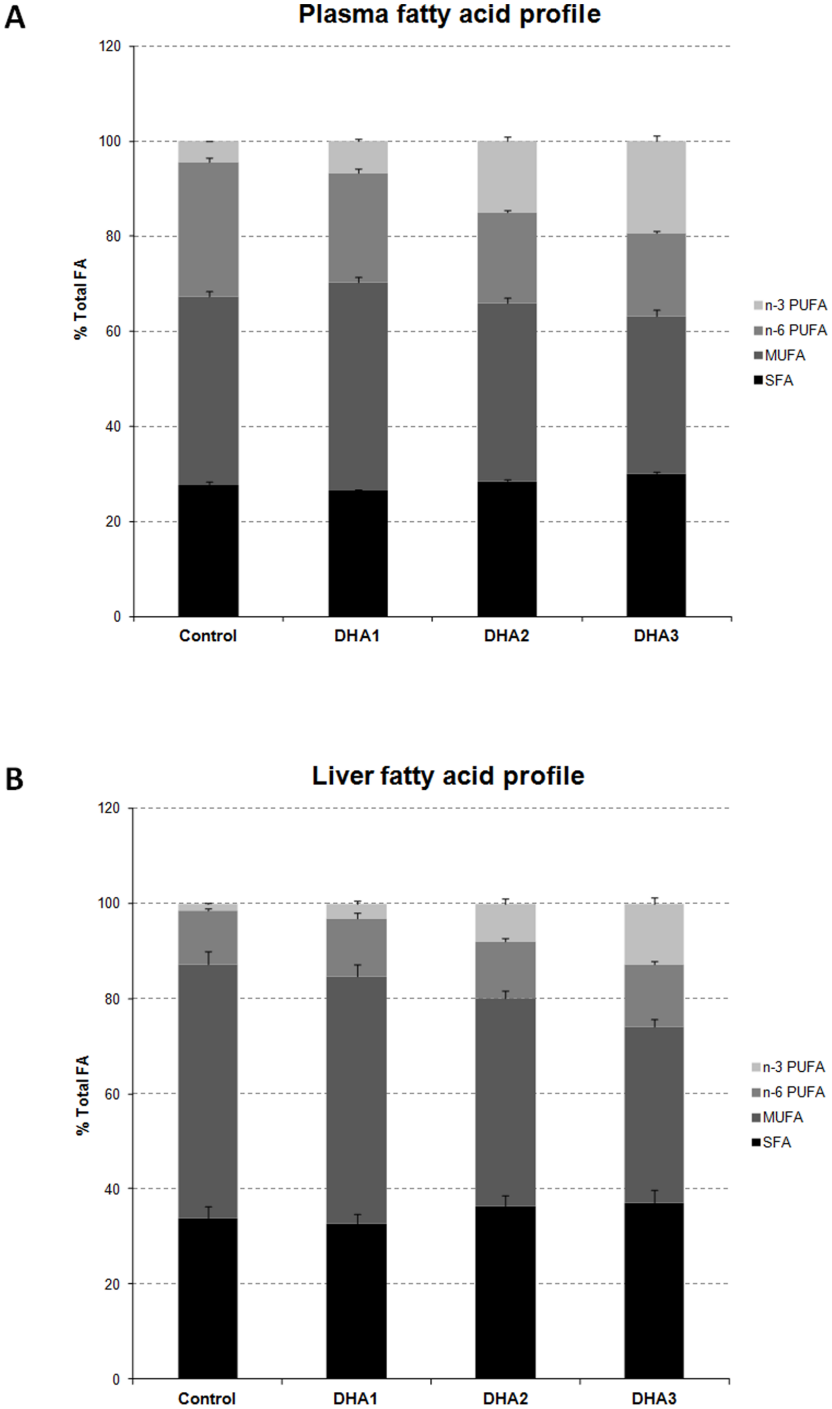
Plasma (A) and liver (B) fatty acid profiles (% total FA). The 4 families of fatty acid (i.e. saturated fatty acids (SFA), monounsaturated fatty acids (MUFA), n-6 PUFA and n-3 PUFA) are represented with different grey shades from black to light grey. Data represent means ± SEM (n = 10/group).

As expected, DHA supplementation produced a substantial enrichment of n-3 PUFAs in both plasma and liver (Figure 3). These changes were dose-dependent in both plasma (R^2^ = 0.95, p<0.01) and liver (R^2^ = 0.99, p<0.01) (Figures S1 & S4 in File S1). Similar changes were observed for plasma and liver levels of DHA (R^2^ = 0.94, p<0.01 and R^2^ = 0.99, p<0.01 respectively, Table S6 and Figures S1 & S4 in File S1) which represents the major n-3 PUFAs in both biological compartments. EPA levels (Table S6 and Figures S1 & S4 in File S1) were also increased in a dose-dependent manner (R^2^ = 0.96, p<0.01 and R^2^ = 0.99, p<0.01). In plasma, n-3 PUFA increases were partially reflected in decreases in n-6 PUFAs, while liver n-6 PUFA composition remained constant. Finally, in both plasma and liver, the MUFA relative abundance was significantly decreased, with the DHA3 group showing the greatest change (−16%, p<0.05 and −31%, p<0.05 respectively) in comparison with the Control group.

### Impact of DHA Supplementation on the Profiles of Plasma Oxylipins and Liver Peroxidized Metabolites

**Figure 4:**
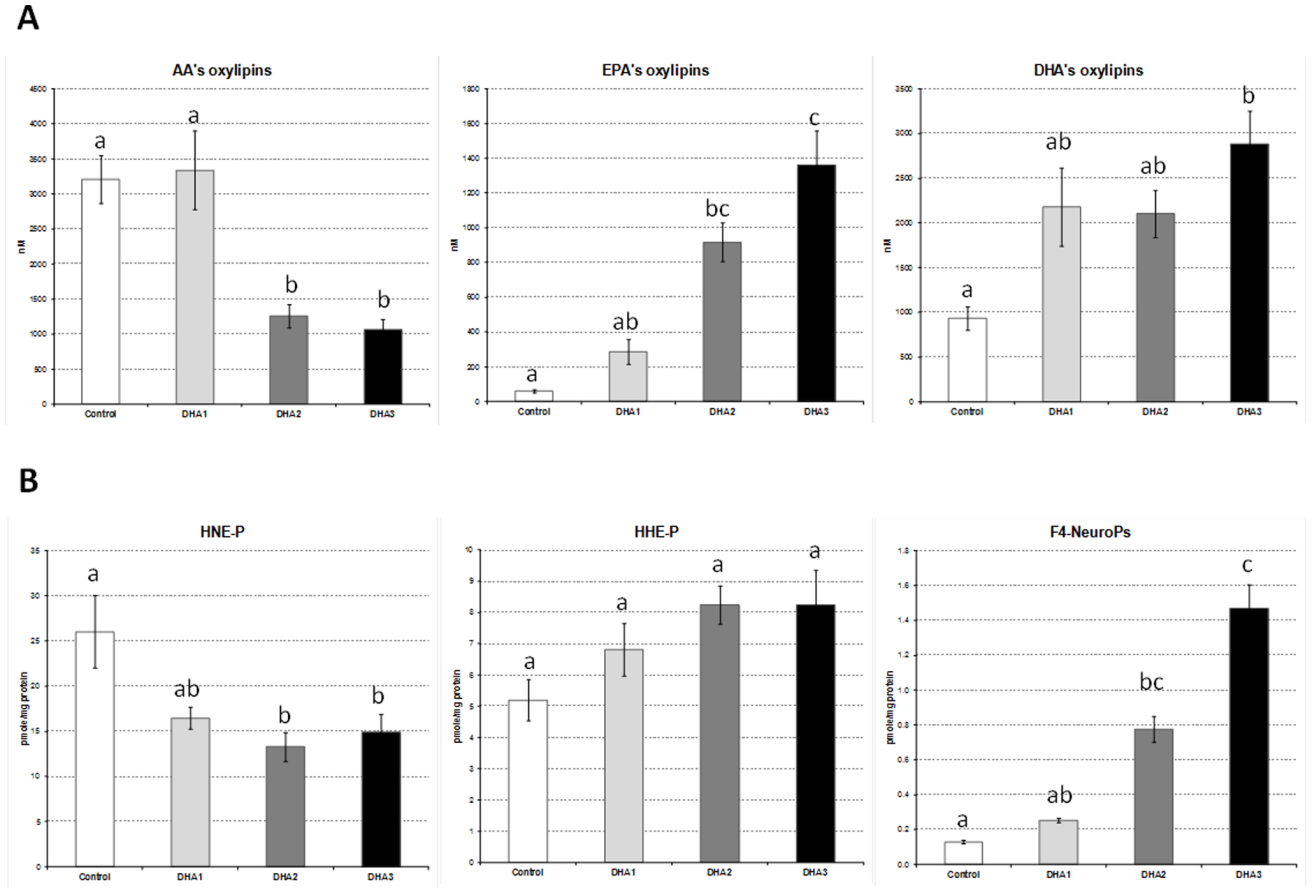
Profiles of PUFAs’s oxygenated metabolites. (A) Plasma concentrations of oxylipins originating from AA (20∶4 n-6), EPA (20∶5 n-3), and DHA (22∶6 n-3). (B) Liver contents of peroxidation metabolites originating from n-6 PUFAs (4-HNE-P), n-3 PUFAs (4-HHE-P) and DHA (F_4_-NeuroPs). Data represent means ± SEM (n = 10/group). ^a,b,c^ Mean values with unlike letters were significantly different (p<0.05).

Plasma and liver profiles of the main oxygenated metabolites originating from the enzymatic and non-enzymatic oxidation of n-6 and n-3 PUFAs are represented in Figure 4 and in Table S7 in File S1. These graphs highlight that DHA supplementation not only modified fatty acids profiles but also influenced the profiles of oxygenated metabolites.

Regarding plasma oxylipins, all families (i.e. AA’s, EPA’s and DHA’s oxylipins, Figure 4A) and sub-families (predominantly represented by epoxides and secondarily by alcohols and diols, Table S7 in File S1) were affected by DHA supplementation. It is interesting to emphasize that the dose-responses differed by oxylipin family leading to different levels of correlations between the plasma content of the parent fatty acids (i.e. AA, EPA and DHA) and their corresponding oxylipins (Figure S1 in File S1). Indeed, the increase of EPA-derived oxylipins in plasma was directly proportional to the dose of DHA and was positively correlated with the plasma level of EPA (R^2^ = 0.99, p<0.01). This latter effect was observed for each individual EPA-derived oxylipin analyzed (i.e. 17(18)-EpETE, 14(15)-EpETE, 17,18-DiHETE, 14,15-DiHETE, 15-HEPE, 12-HEPE, 5-HEPE) (Figure S2 in File S1). The correlation was much weaker between the plasma level of DHA and the DHA-derived oxylipins (R^2^ = 0.74, ns) (Figure S1 in File S1). However, when looking at the individual DHA-derived oxylipins (i.e. 19(20)-EpDPE, 16(17)-EpDPE, 19,20-DiHDPA,17-HDoHE respectively), it should be noticed that the diol and alcohol species (i.e. 19,20-DiHDPA and 17-HDoHE) were actually significantly correlated with plasma DHA concentration (R^2^ = 0.99, p<0.01) (Figure S3 in File S1).

Concerning free radical-mediated peroxidation metabolites, the liver levels of DHA-derived F_4_-NeuroPs were particularly affected by DHA supplementation with concentrations being 2-fold (ns), 6-fold (p<0.05) and 11-fold (p<0.05) increased in the DHA1, DHA2 and DHA3 groups respectively (Figure 4B). This leads to strong positive correlations between F_4_-NeuroPs and liver DHA (Figure S4 in File S1, R^2^ = 0.99, p<0.01). However, the hepatic concentrations of HHE-P, a hydroxyalkenal originating from all n-3 PUFAs, were not significantly different between the groups and correlation between levels of HHE-P and of n-3 PUFAs was not significant (R^2^ = 0.76, ns). Finally, concentrations of HNE-P were reduced in response to DHA supplementation and correlation between liver levels of HNE-P and of n-6 PUFAs was not significant (R^2^ = 0.52, ns). It should be noted that an additional systemic oxidative stress parameter (i.e. oxygen radical absorbance capacity or ORAC) was measured in plasma and no difference appeared between groups (data not shown).

### Predictive Variables of Plaque Area Reduction

**Figure 5:**
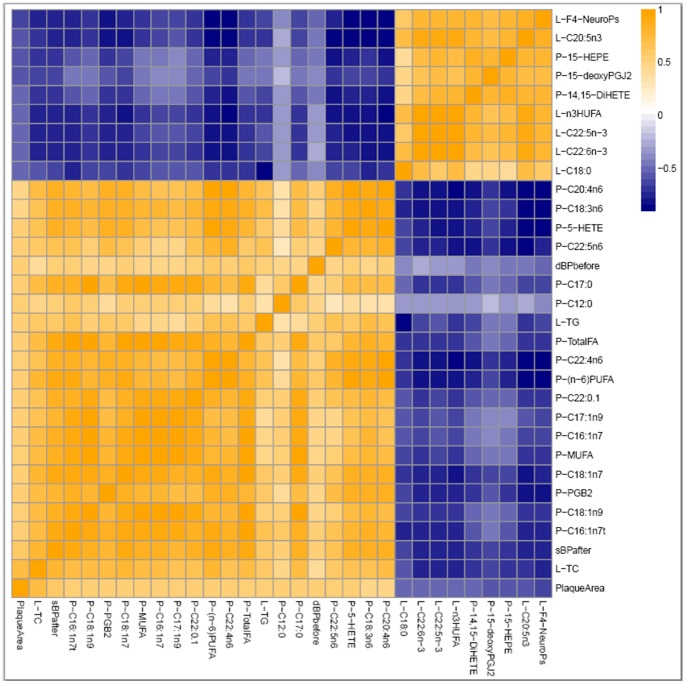
A Spearman’s correlation heatmap. Variables rank from the most positive to most negative correlation with arteriosclerotic plaque area. The displayed variables are the 30 most significantly correlated with arteriosclerotic plaque area at p<0.001 with orange indicating positive and blue indicating negative correlations.

Correlation analyses were used to assess the association of the measured variables with changes in atherosclerotic plaque area. Among the 131 variables assessed, the 30 most significantly correlated with plaque area at p<0.001 are represented in Figure 5. Positive correlations were seen with liver TG and TC, the diet-induced change in sBP and the initial dBP as well as plasma fatty acid and oxylipin levels including several n-6 PUFAs and 5-hydroxyeicosatetraenoic acid (5-HETE), a putative pro-inflammatory mediator derived from 5-lipoxygenase metabolism of AA. Surprisingly, plasma TC and TG did not belong to the 30 most significantly correlated variables. Negative correlations with plaque area first included the liver concentrations of the DHA-derived F_4_-NeuroPs which was also the variable most negatively correlated with plasma TC, a major cardiovascular risk factor (data not shown). It should be noted that the hepatic concentration of F_4_-NeuroPs was also negatively correlated with other cardiovascular risk factors (i.e. liver TC and TG, change in sBP). Interestingly, none of the DHA-derived oxylipins in plasma appeared in the list of the 9 variables most negatively correlated with plaque area. In contrast, liver EPA and two plasma EPA-derived oxylipins (15-HEPE, 14,15-DiHETE) belong to this list. Finally, other variables negatively correlated with plaque area at p<0.001 included the arachidonic acid derived 15-deoxyprostaglandin J_2_ (15-deoxyPGJ_2_) as well as the saturated fatty acid stearate (C18∶0).

**Figure 6:**
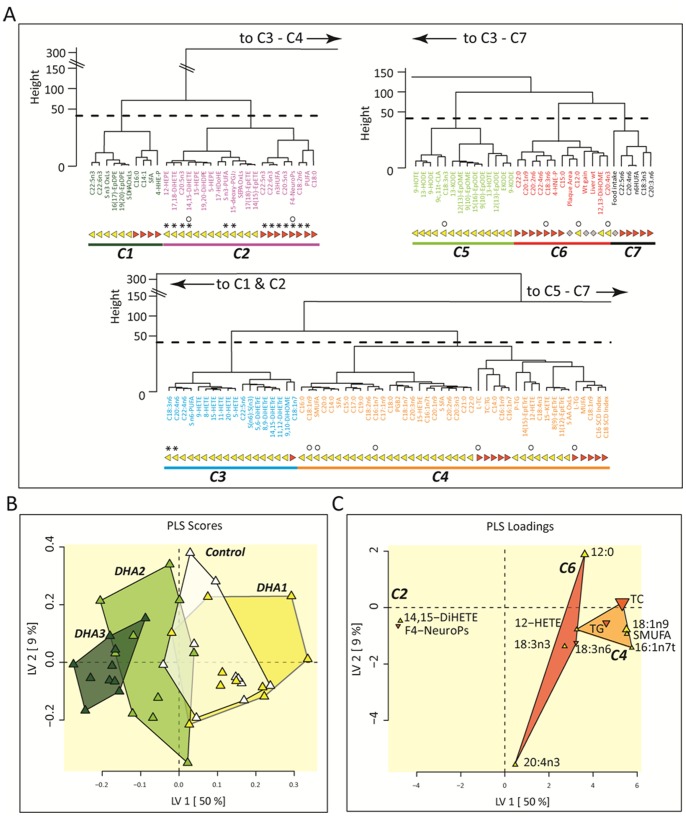
Hierarchical cluster and Partial Least Squares Discrimination Analysis (PLS-DA). (A) Complete data sets (n = 10/group) were segregated into 7 unique clusters of variables (C1–C7) by hierarchical cluster analysis. Clusters were assigned unique colors and used to highlight variables in PLS-DA. Plasma and liver variables are indicated by yellow triangles and orange inverted triangles, respectively. Dominant variables for feeding group discrimination are identified by asterisks (*). Dominant variables for plaque area discrimination are identified by open circles (○). (B) Animals eating each dietary mixture with complete data sets (n = 10/group) were partially segregated by PLS-DA. Mice from DHA1 group were indistinguishable from Controls (p<0.05), while DHA2 (p<0.0001) and DHA3 (p<1.5E-07) were significantly different in this model. (C) Plasma (yellow triangle) and liver (orange triangle) metabolites belonging to clusters C2, C4, and C6 as identified in (A) were included in this model. Predictive variables are labeled with their point size, indicating relative selection frequency of 20–80% in 10 models constructed using a Pearson’s-correlation variable selection filter which out performed other filters in terms of the minimum root mean squared error of prediction (RMSEP = 3.4±1). Analytes appearing in ≥30% of models were retained for the final predictive model construction.

To determine the covariant behavior of the measured variables, a hierarchical cluster analysis and a PLS-DA (Figure 6) were performed to identify variables which best discriminate plaque area or dietary groups (Figure S5 in File S1). Variables were separated into 7 unique variable clusters (Figure 6A). Clusters 1 and 2 were dominated by plasma and liver n-3 PUFAs and n-3 oxylipins, but also contained liver saturated fat, with cluster 2 containing 13 of 15 variables able to segregate the relative dose of the diet groups (see * symbol in Figure 6A). In contrast, clusters 2, 4, 5 and 6 each contained variables responsible for plaque size prediction (see ○ symbol in Figure 6A). It should be noted that the liver F_4_-NeuroPs concentrations appear in both lists of discriminant variables. The PLS discrimination of mice according to their plaque area (Figure 6B) clearly shows the multi-dimensional overlap of the Control and DHA-1 mice, as well as those from the Control and DHA-2 groups, in accordance with the results shown in Figure 2. Moreover, as shown in Figure 6C, groups were discriminated using only 12 of the 152 variables collected, representing 3 variable clusters. Of these, liver F_4_-NeuroPs and the plasma 14,15-diol metabolite of EPA (14,15-DiHETE) were the strongest negative predictors of plaque size, while plasma MUFAs and liver TC were the best positive predictors of plaque size in this LDLR^−/−^ model.

## Discussion

The present study aimed to determine the impact of DHA supplementation on the profiles of PUFA oxygenated metabolites and to investigate their contribution to atherosclerosis prevention. The major findings are as follows: 1) the profiles of both DHA and EPA enzymatic and non-enzymatic metabolites were dose-dependently modified in response to DHA supplementation; 2) F_4_-NeuroPs issued from the free radical-mediated peroxidation of DHA appeared to be a major source of oxygenated metabolites of DHA in the liver during atherogenesis, 3) Hepatic levels of F_4_-NeuroPs were identified as major predictive variables of atherosclerosis prevention.

### DHA Dose-dependently Decreases Atherosclerosis

The dose-response design of the present study not only confirmed the atheroprotective effect of LC n-3 PUFAs previously demonstrated in similar animal models [14],[33],[34] but also showed a strong dose-dependent relationships between the dose of DHA consumed, the atherosclerotic plaque extent, and lipemia. Indeed, more than 90% of the reduction of these cardiovascular risk parameters could be explained by the increased consumption of DHA. Surprisingly, when looking at the Spearman’s correlation heatmap (Figure 5) reporting the 30 most significantly correlated variables with arteriosclerotic plaque, it should be noted that plasma TG and TC do not belong to the list. This suggests that the atheroprotective effects of DHA were in part independent of the reduction of lipemia. This is consistent with results of several animal studies showing that LC n-3 PUFAs can interfere at the vascular level to prevent plaque inception, progression and instability notably by reducing inflammatory processes [34],[35]. Despite the dose-dependent relationships established between the intake of DHA and the reduction of atherosclerotic plaque, the nutritional dose (DHA1 group) showed no effect. This finding is consistent with those of Wang *et al.* [36] which is, to the best of our knowledge, the only other study investigating atheroprotective effects of a nutritional dose of LC n-3 PUFAs in a murine model of atherosclerosis. However, these findings in mice are inconsistent with several human clinical trials showing that modest consumption of fish (i.e., 1–2 servings/wk) reduces significantly coronary death rate and progression of coronary artery atherosclerosis [4],[37].

### DHA Supplementation Strongly Modifies PUFAs Oxygenated Metabolite Profiles

DHA supplementation dose-dependently increased the proportion of LC n-3 PUFAs at the expense of MUFAs and n-6 PUFAs in plasma while in liver only MUFA proportions were reduced. The increases of LC n-3 PUFAs levels were logically allocated to DHA but also to EPA suggesting that retroconversion occurred since no EPA was provided by the diet. Retroconversion of DHA into EPA is a well-described metabolic process which was initially studied in rat hepatocytes and has been reported in several experimental models as well as in humans [38]. Overall, the modifications of plasma and liver fatty acid profiles reported here are consistent with findings in both hypercholesterolemic rabbits [23] and Sprague-Dawley rats [39] given similar dietary levels of DHA. Our experimental design did not allow determining the fatty acid profiles of aorta. However, increased levels of DHA and EPA in aorta [40], heart [39] or macrophages [36] have been reported in rodents given n-3 PUFA doses similar to the present study. Therefore, n-3 PUFA levels in the aorta have probably been enhanced in our model too and the atheroprotective effects obtained are probably in part due to the local action of n-3 PUFAs.

Beside their incorporation into cellular lipids, n-3 PUFAs are susceptible to oxidation by enzymatic or non-enzymatic reactions leading to a large array of metabolites. Enzymatic pathways involve cyclooxygenase and lipooxygenases but also cytochrome P450 epoxygenase and epoxide hydrolase which produce various alcohols, epoxides and diols [7] referred to here as oxylipins. Some of these molecules, namely the alcohols and diols, and additional oxygenated metabolites can arise from the non-enzymatic oxidation of PUFAs by reactive oxygen species and notably include hydroxyalkenals (HNE and HHE from n-6 and n-3 PUFAs respectively) and F_2_-IsoPs/F_4_-NeuroPs (from AA and DHA respectively) [9],[10],[11]. Little is known about the impact of n-3 PUFA supplementation on the profiles of their specific oxygenated metabolites. Moreover, to the best of our knowledge, no study has reported so far the profile of enzymatic and non-enzymatic oxygenated metabolites of n-3 PUFAs in a model of atherosclerosis. Though, this could be particularly relevant since both the enzymatic and non-enzymatic pathways are known to be enhanced during atherogenesis [12],[41] and high oxylipins levels have been reported in ApoE^-/−^ mice [42]. Concerning the enzymatic metabolites, DHA supplementation induced substantial modifications of their plasma profiles with a reduction of the AA oxylipins and increases of EPA and DHA oxylipins. Interestingly, the impact on EPA oxylipins was higher than on DHA oxylipins probably because of the higher change in their precursor fatty acids. In healthy humans given EPA+DHA supplement (∼1.4% daily energy intake, i.e. close to DHA2 group) for 4 weeks [7], similar modifications of the oxylipins profiles were reported, but to a lesser extent than ours, especially for the effects on AA and EPA oxylipins. Inter-individual variability within the study cohort likely reduced the average efficacy reported in this human intervention study [43]. Concerning the dose-response relationships, it should be emphasized that the present data reveal different levels of correlation between EPA and DHA with their corresponding oxylipins. Indeed, the plasma levels of EPA oxylipins were strongly and positively correlated with plasma EPA concentration as previously shown in human [44]. This effect that was less pronounced for DHA and its corresponding oxylipins when considering the entire family of oxylipins. However, when looking at the individual DHA-derived oxylipins, correlations were much stronger with diol and alcohol species (i.e. 19,20-DiHDPA and 17-HDoHE) which are actually produced both by enzymatic and non-enzymatic pathways. Plasma DHA also showed strong positive correlations with liver F_4_-NeuroP levels suggesting that DHA was readily peroxidized. This is consistent with previous findings in LDLR^−/−^ mice [33] exposed to LC n-3 PUFAs (34 mg of EPA +23 mg of DHA/day/mice, i.e. ∼3% daily energy intake) for 12 weeks, which showed 5-fold and 3-fold increased F_4_-NeuroPs levels in the liver and heart respectively. This suggests that a proportion of DHA incorporated in tissue lipids is readily peroxidized and F_4_-NeuroPs is likely to be a relevant biomarker of this non-enzymatic oxidation pathway. In this study, we reported a maximal liver F_4_-NeuroP increase of 11-fold with the highest dose (35.5 mg/day/mice) DHA3 group *vs.* Control. These results together with other studies [33] thus demonstrate that DHA supplementation of atherosclerotic mice induces a dose-dependent increase of F_4_-NeuroPs in peripheral tissues, and/or cell types that accumulate DHA such as aorta [40] and immune cells [36].

### F_4_-NeuroPs as a Major Predictive Variable of Atherosclerosis Prevention

The analyses of the relationships between plaque extent and the other variables measured using correlation analyses, hierarchical cluster and PLS-DA indicated that among the ∼131 variables measured, the liver content of DHA-derived F_4_-NeuroPs was the most negatively correlated variable with plaque extent. Moreover, when a correlation strength filter was used for variable selection in predictive model production, the F_4_-NeuroPs was one of just two predictive variables needed to predict atherosclerosis prevention. This strongly reinforces the initial hypothesis suggesting that peroxidized metabolites of DHA could contribute to its atheroprotective effects and suggests that F_4_-NeuroPs could be relevant candidates. However, it must be emphasized that these are correlative associations, and causal linkage remains to be further assessed. Moreover, it cannot be excluded that other lipid mediators could have contributed to the reduction of atherosclerosis. For instance, human atherosclerotic lesions have been shown to over express 12- and 15-lipoxygenases [45] and DHA-dependent impacts on cardiovascular disease are influenced by the activity of these enzymes [46]. It should also be emphasized that despite an absence of EPA intake, the current results revealed that the levels of EPA and its corresponding oxylipins were substantially increased by DHA supplementation. Moreover, the level of 14,15-DiHETE, a stable metabolite of the EPA and a cytochrome P450 product, showed a negative correlation with plaque size. All together, these data suggest that F_4_-NeuroPs are putative contributors of atherosclerosis prevention in coordination with other lipid mediators.

The present study is to the best of our knowledge, the first one to establish *in vivo* an inverse dose-dependent relationship between the productions of DHA-derived peroxidized metabolites and atherosclerosis development. Nevertheless, our results are consistent with several lines of evidence demonstrated *in vitro*. Indeed, the group of Sethi *et al.* [47] demonstrated that oxidized EPA and DHA (obtained by *ex-vivo* oxidation of the native fatty acids with CuSO_4_ and ascorbic acid) were able to reduce adhesion of U937 monocyte cells to endothelial cells and decrease the expression of adhesion molecules whereas native EPA and DHA had no effect. The bioactive oxygenated metabolites issued from EPA and DHA were not identified but the authors clearly showed that the non-enzymatic oxidation of EPA and DHA was a mandatory prerequisite to make them bioactive on endothelial cells. The reduction of adhesion molecules expression was achieved through the inhibition of NFκB binding activity and was latter associated with a PPARγ dependent mechanism [48]. Equally, it has been nicely shown that peroxidation of DHA was responsible for its effects on transient outward current and steady-state outward current in rat ventricular myocytes [49]. The group of Morrow and Roberts, pioneers in the *in vivo* identification of IsoP/NeuroPs [10],[50] substantially contributed to this field of research by demonstrating that cyclopentenone A4/J4-NeuroPs issued from the peroxidation of DHA were anti-inflammatory mediators in the RAW267.4 murine macrophage cell line [51]. Similar results were reported recently with 15-A_3t_-IsoPs [52], a specific peroxidized metabolite of EPA. These two studies confirmed the inhibition of the NFκB pathway as a major mechanism of action of EPA and DHA peroxidized metabolites. Finally, even though the *in vivo* study published by Saraswathi *et al.* [33] did not investigate the relationship between the production of F_4_-NeuroPs and atherosclerosis regression, the authors did speculate that the F_4_-NeuroPs could play a role in the prevention of atherosclerosis. All together, these results challenge the long standing paradigm suggesting that peroxidized metabolites of PUFAs might only be cytotoxic molecules.

In conclusion, the present study showed that supplementation with DHA during atherogenesis is associated with the production of an array of oxygenated metabolites in association with reduced atherosclerosis progression. Among them, the F_4_-NeuroPs arising from the peroxidation of DHA were found to be a new potentially relevant biomarker of DHA exposure and one of the best predictive variables of atherosclerosis prevention in the LDLR^−/−^ mouse. Further investigations are needed to determine if F_4_-Neuroprostanes can contribute to the anti-atherogenic effects of DHA and decipher their molecular mechanisms of action. This will help to elucidate novel interactions between lipid peroxidation metabolites and atherosclerosis.

## Supporting Information

File S1 **Figure S1, Correlations between doses of DHA given to LDLR^-/-^ mice and plasma levels of PUFA and of AA, EPA, and DHA and their corresponding oxylipins.** **Figure S2, Correlations between plasma levels of EPA, and their corresponding specific oxylipins.** **Figure S3, Correlations between plasma levels of DHA, and their corresponding specific oxylipins**. **Figure S4, Correlations between doses of DHA given to LDLR^-/-^ mice and liver levels of PUFA and plasma levels of n-6 PUFA, n-3 PUFA and DHA and their corresponding peroxidized metabolites**. **Figure S5, Partial least squares discrimination analysis of dietary groups in LDLR^-/-^ given by daily oral gavages increasing doses of DHA.** (A) Animals eating each dietary mixture with complete data sets (n = 10/group) were segregated by PLS analyses. (B) Group segregation was driven by plasma (yellow triangle) and liver (orange triangle) metabolites belonging to clusters C2, C3, and C4 as identified in Figure 7. The plasma EPA metabolite, 17,18-dihydroxyeicosaotetraenoic acid (17,18-DiHETE) and the liver DHA metabolite group F4-neuroprostanes (F4-NeuroPs) were the most frequently selected variables occurring in 80% and 60% of models, respectively. **Figure S6, Correlation between plasma F2-isoprostanes and 9-HETE levels**. **Figure S7, Correlations between plasma oxylipins and plasma 9-HETE levels**. **Table S1, Surrogates recoveries**. **Table S2, Oxylipin assay UPLC solvent gradient**. **Table S3, UPLC/MS-MS parameters of metabolites measured in plasma**. **Table S4,** Concentrations of plasma* and liver^†^ triglycerides (TG) and total cholesterol (TC). Table S5, **Changes in systolic and diastolic blood pressures (sBP and dBP), and heart rate (HR)**. **Table S6,** **Plasma and liver levels of major polyunsaturated fatty acids (PUFA)**. LDLR^-/-^ mice were given by daily oral gavages (20 weeks) either oleic acid rich sunflower oil (Control group) or a mixture of oleic acid rich sunflower oil and DHA rich tuna oil providing 0.1%, 1% or 2% of energy as DHA (DHA1, DHA2, and DHA3 groups respectively). **Table S7,** **Plasma levels of PUFAs-derived oxylipins.** LDLR^-/-^ mice were given by daily oral gavages (20 weeks) either oleic acid rich sunflower oil (Control group) or a mixture of oleic acid rich sunflower oil and DHA rich tuna oil providing 0.1%, 1% or 2% of energy as DHA (DHA1, DHA2, and DHA3 groups respectively). **Table S8, Thromboxane and prostaglandin stability through sample processing**. **Table S9, Fatty acid triol stability through sample processing**. **Table S10, Fatty acid diol stability through sample processing.**
(DOCX)
