# Supplementary material for: Lipid Profiling following Intake of the Omega 3 Fatty Acid DHA Identifies the Peroxidized Metabolites F4-Neuroprostanes as the Best Predictors of Atherosclerosis Prevention
Source: PLoS One. 2014 Feb 18;9(2):e89393. doi: 10.1371/journal.pone.0089393 (PMC3928438; doi:10.1371/journal.pone.0089393)
Supplement: File S1 — Figure S1, Correlations between doses of DHA given to LDLR-/- mice and plasma levels of PUFA and of AA, EPA, and DHA and their corresponding oxylipins. Figure S2, Correlations between plasma levels of EPA, and their corresponding specific oxylipins. Figure S3, Correlations between plasma levels of DHA, and their corresponding specific oxylipins. Figure S4, Correlations between doses of DHA given to LDLR-/- mice and liver levels of PUFA and plasma levels of n-6 PUFA, n-3 PUFA and DHA and their corresponding peroxidized metabolites. Figure S5, Partial least squares discrimination analysis of dietary groups in LDLR-/- given by daily oral gavages increasing doses of DHA. (A) Animals eating each dietary mixture with complete data sets (n = 10/group) were segregated by PLS analyses. (B) Group segregation was driven by plasma (yellow triangle) and liver (orange triangle) metabolites belonging to clusters C2, C3, and C4 as identified in Figure 7. The plasma EPA metabolite, 17,18-dihydroxyeicosaotetraenoic acid (17,18-DiHETE) and the liver DHA metabolite group F4-neuroprostanes (F4-NeuroPs) were the most frequently selected variables occurring in 80% and 60% of models, respectively. Figure S6, Correlation between plasma F2-isoprostanes and 9-HETE levels. Figure S7, Correlations between plasma oxylipins and plasma 9-HETE levels. Table S1, Surrogates recoveries. Table S2, Oxylipin assay UPLC solvent gradient. Table S3, UPLC/MS-MS parameters of metabolites measured in plasma. Table S4, Concentrations of plasma* and liver† triglycerides (TG) and total cholesterol (TC). Table S5, Changes in systolic and diastolic blood pressures (sBP and dBP), and heart rate (HR). Table S6, Plasma and liver levels of major polyunsaturated fatty acids (PUFA). LDLR-/- mice were given by daily oral gavages (20 weeks) either oleic acid rich sunflower oil (Control group) or a mixture of oleic acid rich sunflower oil and DHA rich tuna oil providing 0.1%, 1% or 2% of energy as DHA (DHA1, DHA2, and DHA3 [file pone.0089393.s001.docx]

**Table S1**. **Surrogates recoveries**

| **Compound** | **Recoveries**  **(%)** |
| --- | --- |
| **Methylation Efficiency** | |
| 15:1n5 | 80.4 ± 12% |
| **Extraction and GC-MS Analysis Surrogates** | |
| d31-16:0-TG | 75.1 ± 17% |
| d35-18:0-PC | 75.1 ± 15% |
| 22:1n9-CE | 75.5 ± 17% |
| 22:3n3 | 75.5 ± 13% |
| **Hydrolysis Control, SPE Isolation &  LC-MS/MS Analysis Surrogates** | |
| d4-6-keto-PGF1a | 62.1 ± 6.2 |
| d4-TXB2 | ND |
| d4-PGF2a | 55.1 ± 6.9 |
| d4-PGD2 | ND |
| d3-LTE4 | ND |
| d4-LTB4 | 87.5 ± 13 |
| d11-14,15-DiHETrE | 91.2 ± 12 |
| d6-20-HETE | 48.7 ± 15 |
| d4-9-HODE | 75.1 ± 13 |
| d8-12-HETE | 59.1 ± 8.9 |
| d8-5-HETE | 90.8 ± 24 |
| d4-12(13)-EpOME | 35.4 ± 10 |

**Table S2. Oxylipin assay UPLC solvent gradient**

| **Time (min)** | **Solvent A (%)** |
| --- | --- |
| 0 | 75 |
| 1 | 60 |
| 2.5 | 58 |
| 4.5 | 50 |
| 10.5 | 35 |
| 12.5 | 25 |
| 14 | 15 |
| 14.5 | 5 |
| 15 | 75 |
| 16 | 75 |

Solvent A = 0.1% acetic acid

Solvent B = 90:10 v/v acetonitrile/isopropanaol

**Table S3. UPLC/MS-MS parameters of metabolites measured in plasma**

| **Compound** | **tR (min)** | **Acquisition Period** | **Precursor (*m*/*z*)** | **Product (*m*/*z*)** | **Dwell Time (msec)** | **DCP (V)** | **CE (V)** | **IS/ Surrogate** | **IDL***^a^* **(nM)** |
| --- | --- | --- | --- | --- | --- | --- | --- | --- | --- |
|  |  |  |  |  |  |  |  |  |  |
| PHAU | 3.34 | 1 | 249.2 | 130.1 | 10 | -45 | -20 |  | IS |
| 20-carboxy-LTB4 | 3.58 | 1 | 365.3 | 347.2 | 10 | -70 | -25 | d4 6-keto PGF1a | 3.54 |
| Resolvin E1 | 3.71 | 1 | 349.3 | 195.2 | 50 | -70 | -25 | d4 6-keto PGF1a | 0.195 |
| 6-keto-PGF1a | 3.71 | 1 | 369.3 | 163.1 | 10 | -70 | -40 | d4 6-keto PGF1a | 0.692 |
| d4 6-keto PGF1a | 3.71 | 1 | 373.3 | 167.1 | 10 | -60 | -40 | PHAU | IS |
| 20-hydroxy-LTB4 | 3.8 | 1 | 351.3 | 195.2 | 10 | -65 | -25 | d4 6-keto PGF1a | 0.58 |
| PGE3 | 4.53 | 2 | 349.3 | 269.2 | 10 | -45 | -22 | d4-PGD2a | --- |
| d4-TXB2 | 4.52 | 2 | 373.3 | 173.1 | 10 | -50 | -25 | PHAU | IS, --- |
| TXB2 | 4.54 | 2 | 369.3 | 169.1 | 10 | -50 | -25 | d4-TXB2 | --- |
| 9,12,13-TriHOME | 5 | 2 | 329.2 | 211.2 | 10 | -70 | -32 | d4-PGF2a | 0.704 |
| d4-PGF2a | 5.06 | 2 | 357.3 | 197.2 | 10 | -65 | -35 | PHAU | IS |
| PGF2a / F2-IsoPs | 5.07 | 2 | 353.3 | 193.2 | 10 | -65 | -35 | d4-PGF2a | 0.749 |
| 9,10,13-TriHOME | 5.11 | 2 | 329.2 | 171.1 | 10 | -70 | -30 | d4-PGF2a | 0.903 |
| PGE2 | 5.28 | 2 | 351.3 | 271.2 | 10 | -35 | -25 | d4-PGD2a | --- |
| PGE1 | 5.36 | 2 | 353.3 | 317.2 | 10 | -50 | -22 | d4-PGD2a | --- |
| d4-PGD2 | 5.49 | 2 | 355.3 | 275.2 | 10 | -45 | -26 | PHAU | IS, --- |
| PGD2 | 5.62 | 2 | 351.3 | 271.2 | 10 | -35 | -25 | d4-PGD2a | --- |
| Resolvin D1 | 6.12 | 3 | 375.3 | 121.1 | 50 | -50 | -40 | d4-PGF2a | 0.301 |
| 11,12,15 THET | 6.03 | 3 | 353.3 | 167.1 | 50 | -60 | -30 | d4-PGF2a | 0.0848 |
| Lipoxin A4 | 6.22 | 3 | 351.3 | 217.2 | 50 | -50 | -26 | d4-PGF2a | 0.335 |

*^a^* IDL = Lowest detectable level in plasma nM equivalents; Alkali unstable (---); uncalibrated data collection (UnCal); Internal Standards (IS)

**Table S3. UPLC/MS-MS parameters of metabolites measured in plasma (continued)**

| **Compound** | **tR (min)** | **Acquisition Period** | **Precursor (*m*/*z*)** | **Product (*m*/*z*)** | **Dwell Time (msec)** | **DCP (V)** | **CE (V)** | **IS/ Surrogate** | **IDL***^a^* **(nM)** |
| --- | --- | --- | --- | --- | --- | --- | --- | --- | --- |
| PGJ2/ δ 12-PGJ2 | 7.13 | 4 | 333.2 | 233.2 | 5 | -52 | -14 | d4-PGF2a | --- |
| PGB2 | 7.11 | 4 | 333.3 | 235.2/175.1 | 5 | -74 | -28 | d4-PGF2a | --- |
| LTB5 | 7.35 | 4 | 333.3 | 195.2 | 5 | -70 | -20 | d4-PGF2a | 0.505 |
| DiHETHF | 7.41 | 4 | 353.3 | 127.1 | 30 | -55 | -35 | d11-14,15-DiHETrE | --- |
| 15,16-DiHODE | 7.69 | 5 | 311.2 | 235.2 | 10 | -65 | -22 | d11-14,15-DiHETrE | 1.68 |
| 12,13-DiHODE | 7.79 | 5 | 311.2 | 183.2 | 10 | -70 | -30 | d11-14,15-DiHETrE | 1.69 |
| 8,15-DiHETE | 7.8 | 5 | 335.3 | 235.2 | 10 | -65 | -22 | d11-14,15-DiHETrE | 0.994 |
| Hepoxilin A3 | 7.81 | 5 | 335.2 | 171.1 | 50 | -85 | -20 | d11-14,15-DiHETrE | --- |
| 9,10-DiHODE | 7.83 | 5 | 311.2 | 201.2 | 10 | -65 | -30 | d11-14,15-DiHETrE | 1.42 |
| d3-LTE4 | 7.88 | 5 | 441.4 | 336.3 | 50 | -80 | -30 | CUDA | IS, --- |
| LTE4 | 7.91 | 5 | 438.4 | 333.3 | 50 | -60 | -25 | d3-LTE4 | --- |
| 17,18-DiHETE | 8.08 | 5 | 335.3 | 247.2 | 10 | -60 | -25 | d11-14,15-DiHETrE | 0.377 |
| 5,15-DiHETE | 8.15 | 5 | 335.3 | 173.1 | 10 | -45 | -21 | d11-14,15-DiHETrE | 0.304 |
| 6-trans-LTB4 | 8.26 | 5 | 335.3 | 195.2 | 20 | -70 | -21 | d4-LTB4 | 0.601 |
| 14,15-DiHETE | 8.42 | 5 | 335.3 | 207.2 | 10 | -55 | -25 | d11-14,15-DiHETrE | 0.322 |
| CUDA | 8.47 | 5 | 339.4 | 214.2 | 10 | -65 | -35 |  | IS |
| d4-LTB4 | 8.49 | 5 | 339.3 | 163.1 | 20 | -70 | -38 | CUDA | IS |
| LTB4 | 8.6 | 5 | 335.3 | 195.2 | 10 | -70 | -21 | d4-LTB4 | 0.654 |
| 12,13-DiHOME | 8.81 | 6 | 313.3 | 183.2 | 5 | -70 | -30 | d11-14,15-DiHETrE | 1.69 |
| 10,11-DHHep | 8.9 | 6 | 301.2 | 283.2 | 10 | -70 | -30 | CUDA | IS |
| 9,10-DiHOME | 9.23 | 6 | 313.3 | 201.2 | 5 | -70 | -30 | d11-14,15-DiHETrE | 1.01 |
| d11-14,15-DiHETrE | 9.39 | 6 | 348.4 | 207.2 | 10 | -64 | -28 | CUDA | IS |
| LTD4 | 9.41 | 6 | 495.45 | 177.1 | 50 | -70 | -25 | d3-LTE4 | --- |

*^a^* IDL = Lowest detectable level in plasma nM equivalents; Alkali unstable (---); uncalibrated data collection (UnCal); Internal Standards (IS)

**Table S3. UPLC/MS-MS parameters of metabolites measured in plasma (continued)**

| **Compound** | **tR (min)** | **Acquisition Period** | **Precursor (*m*/*z*)** | **Product (*m*/*z*)** | **Dwell Time (msec)** | **DCP (V)** | **CE (V)** | **IS/ Surrogate** | **IDL***^a^* **(nM)** |
| --- | --- | --- | --- | --- | --- | --- | --- | --- | --- |
| 19,20-DiHDoPE | 9.44 | 6 | 361.3 | 273.2 | 10 | -74 | -24 | d11-14,15-DiHETrE | 1.22 |
| 14,15-DiHETrE | 9.48 | 6 | 337.3 | 207.2 | 10 | -65 | -25 | d11-14,15-DiHETrE | 0.737 |
| 11,12-DiHETrE | 10.06 | 7 | 337.3 | 167.1 | 10 | -60 | -27 | d11-14,15-DiHETrE | 0.753 |
| 9-HOTE | 10.32 | 7 | 293.2 | 171.1 | 10 | -65 | -22 | d4-9-HODE | 0.245 |
| 12(13)-Ep-9-KODE | 10.33 | 7 | 309.2 | 291.2 | 10 | -65 | -20 | d4-9-HODE | 0.261 |
| 13-HOTE | 10.44 | 7 | 293.2 | 195.2 | 10 | -65 | -25 | d4-9-HODE | 0.416 |
| 8,9-DiHETrE | 10.61 | 7 | 337.3 | 127.1 | 10 | -55 | -30 | d11-14,15-DiHETrE | 0.659 |
| 15-deoxy PGJ2 | 10.68 | 7 | 315.2 | 271.2 | 10 | -60 | -20 | d11-14,15-DiHETrE | 0.211 |
| d6-20-HETE | 10.86 | 7 | 325.3 | 281.2 | 10 | -70 | -25 | CUDA | IS |
| 15-HEPE | 10.88 | 7 | 317.2 | 219.2 | 10 | -60 | -20 | d8-12-HETE | 1.79 |
| 20-HETE | 10.91 | 7 | 319.2 | 275.2 | 10 | -65 | -24 | d6-20-HETE | 1.22 |
| 12-HEPE | 11.23 | 7 | 317.2 | 179.1 | 10 | -60 | -20 | d8-12-HETE | 1.83 |
| 5,6-DiHETrE | 11.38 | 7 | 337.3 | 145.1 | 10 | -70 | -25 | d11-14,15-DiHETrE | 0.633 |
| 13-HODE | 11.84 | 8 | 295.2 | 195.2 | 30 | -65 | -25 | d4-9-HODE | 3.43 |
| 5-HEPE | 11.84 | 8 | 317.2 | 115.1 | 10 | -60 | -20 | d8-5-HETE | 1.19 |
| d4-9-HODE | 11.89 | 8 | 299.2 | 172.1 | 10 | -70 | -26 | CUDA | IS |
| 9-HODE | 11.95 | 8 | 295.2 | 171.1 | 10 | -60 | -25 | d4-9-HODE | 2.17 |
| 15(16)-EpODE | 12.02 | 8 | 293.2 | 275.2 | 50 | -60 | -20 | d4-12(13)-EpOME | 0.505 |
| 17(18)-EpETE | 12.11 | 8 | 317.2 | 259.2 | 30 | -55 | -15 | d4-12(13)-EpOME | 0.688 |
| 15-HETE | 12.13 | 8 | 319.2 | 219.2 | 50 | -70 | -16 | d8-12-HETE | 0.313 |
| 13-KODE | 12.21 | 8 | 293.2 | 179.1 | 10 | -70 | -26 | d4-9-HODE | 0.554 |
| 15-HpETE | 12.21 | 8 | 335.2 | 113.1 | 10 | -58 | -20 | d8-12-HETE | UnCal |
| 9(10)-EpODE | 12.22 | 8 | 293.2 | 275.2 | 10 | -60 | -20 | d4-12(13)-EpOME | 0.164 |
| 17-HDoHE | 12.22 | 8 | 343.3 | 281.2 | 10 | -45 | -20 | d8-12-HETE | 0.871 |
| 13-HpODE | 12.41 | 8 | 311.2 | 179.1 | 10 | -40 | -20 | d4-9-HODE | UnCal |

*^a^* IDL = Lowest detectable level in plasma nM equivalents; Alkali unstable (---); uncalibrated data collection (UnCal); Internal Standards (IS)

**Table S3. UPLC/MS-MS parameters of metabolites measured in plasma (continued)**

| **Compound** | **tR (min)** | **Acquisition Period** | **Precursor (*m*/*z*)** | **Product (*m*/*z*)** | **Dwell Time (msec)** | **DCP (V)** | **CE (V)** | **IS/ Surrogate** | **IDL***^a^* **(nM)** |
| --- | --- | --- | --- | --- | --- | --- | --- | --- | --- |
| 12(13)-EpODE | 12.42 | 8 | 293.2 | 183.2 | 10 | -60 | -25 | d4-12(13)-EpOME | 0.373 |
| 15-KETE | 12.48 | 8 | 317.2 | 273.2 | 10 | -65 | -20 | d8-12-HETE | 4.98 |
| 11-HETE | 12.57 | 8 | 319.2 | 167.1 | 30 | -55 | -15 | d8-12-HETE | 1.08 |
| 14(15)-EpETE | 12.59 | 8 | 317.2 | 247.2 | 30 | -45 | -15 | d4-12(13)-EpOME | 0.493 |
| 9-KODE | 12.66 | 8 | 293.2 | 185.2 | 10 | -70 | -30 | d4-9-HODE | 3.74 |
| d8-12-HETE | 12.71 | 8 | 327.2 | 184.2 | 10 | -60 | -21 | CUDA | IS |
| 9-HpODE | 12.79 | 8 | 311.2 | 185.2 | 10 | -40 | -20 | d4-9-HODE | UnCal |
| 12-HETE | 12.82 | 8 | 319.2 | 179.1 | 10 | -60 | -21 | d8-12-HETE | 2.01 |
| 8-HETE | 12.96 | 8 | 319.2 | 155.1 | 10 | -55 | -21 | d8-12-HETE | 1.94 |
| 12-HpETE | 12.98 | 8 | 335.2 | 153.1 | 10 | -58 | -20 | d8-12-HETE | UnCal |
| 15-HETrE | 13.02 | 8 | 321.2 | 221.2 | 10 | -65 | -24 | d8-12-HETE | 0.712 |
| 12-KETE | 13.07 | 8 | 317.2 | 273.2 | 10 | -65 | -20 | d8-12-HETE | UnCal |
| 9-HETE | 13.16 | 8 | 319.2 | 167.1 | 30 | -55 | -15 | d8-12-HETE | 1.94 |
| d8-5-HETE | 13.4 | 8 | 327.2 | 116.1 | 10 | -55 | -22 | CUDA | IS |
| 19(20)-EpDoPE | 13.46 | 8 | 343.3 | 281.2 | 10 | -45 | -20 | d4-12(13)-EpOME | 1.67 |
| 5-HETE | 13.5 | 8 | 319.2 | 257.2 / 115.1 | 10 | -50 | -20 | d8-5-HETE | 1.78 |
| d4-12(13)-EpOME | 13.57 | 8 | 299.2 | 198.1 | 10 | -65 | -25 | CUDA | IS |
| 12(13)-EpOME | 13.65 | 8 | 295.2 | 195.1 | 10 | -65 | -25 | d4-12(13)-EpOME | 0.375 |
| 14(15)-EpETrE | 13.73 | 8 | 319.2 | 219.2 | 10 | -70 | -16 | d4-12(13)-EpOME | 4.61 |
| 16(17)-EpDoPE | 13.84 | 8 | 343.5 | 273.5 | 30 | -55 | -15 | d4-12(13)-EpOME | 1.4 |
| 9(10)-EpOME | 13.88 | 8 | 295.2 | 171.1 | 10 | -60 | -25 | d4-12(13)-EpOME | 0.359 |
| 5-HpETE | 14.06 | 8 | 335.2 | 155.1 | 10 | -58 | -20 | d8-12-HETE | UnCal |
| 5-KETE | 14.16 | 8 | 317.2 | 203.2 | 50 | -70 | -25 | d8-5-HETE | 0.433 |
| 11(12)-EpETrE | 14.2 | 8 | 319.2 | 167.1 | 30 | -55 | -15 | d4-12(13)-EpOME | 3.91 |
| 8(9)-EpETrE | 14.4 | 8 | 319.2 | 155.1/ 167.1 | 10 | -55 | -15 | d4-12(13)-EpOME | 4.54 |

*^a^* IDL = Lowest detectable level in plasma nM equivalents; Alkali unstable (---); uncalibrated data collection (UnCal); Internal Standards (IS)

**Table S4**. **Concentrations of plasma* and liver**^†^ **triglycerides (TG) and total cholesterol (TC)**.

| Lipid Parameters | **Control** | **DHA1** | **DHA2** | **DHA3** | **P value** |
| --- | --- | --- | --- | --- | --- |
| Plasma TG (mM) | 1.11 ± 0.12 ^a^ | 1.10 ± 0.12 ^ab^ | 0.84 ± 0.09 ^ab^ | 0.70 ± 0.07 ^b^ | 0.015 |
| Plasma TC (mM) | 13.20 ± 0.54 ^ab^ | 13.85 ± 0.47 ^a^ | 11.16 ± 0.56 ^bc^ | 9.45 ± 0.30 ^c^ | <0.001 |
| Liver TG (µg/mg) | 102.62 ± 14.63 ^a^ | 91.09 ± 11.53 ^a^ | 55.18 ± 5.10 ^ab^ | 45.61 ± 6.60 ^b^ | 0.002 |
| Liver TC (µg/mg) | 7.15 ± 0.60 ^a^ | 7.98 ± 0.57 ^a^ | 5.21 ± 0.49 ^ab^ | 3.80 ± 0.20 ^b^ | <0.001 |

All data are represented as mean ± SEM, *: n=30/group for plasma TG and TC; † n=10/group for liver TG and TC. a,b,c Mean values with unlike letters were significantly different (p < 0.05).

**Table S5**. **Changes in systolic and diastolic blood pressures (sBP and dBP), and heart rate (HR)**.

|  | **Control** | | **DHA1** | | **DHA2** | | **DHA3** | | **P value*** |
| --- | --- | --- | --- | --- | --- | --- | --- | --- | --- |
|  | Before | After | Before | After | Before | After | Before | After |  |
| Change in sBP (mmHg) | 98 ± 2 | 97 ± 2 ^a^ | 100 ± 3 | 99 ± 2 ^a^ | 99 ± 2 | 96 ± 2 ^a^ | 101 ± 2 | 85 ± 2 ^b^ | 0.002 |
| Change in dBP (mmHg) | 67 ± 2 | 72 ± 2 | 68 ± 4 | 73 ± 2 | 66 ± 2 | 72± 1 | 67 ± 3 | 62 ± 2 | 0.181 |
| Change in HR (bpm) | 551 ± 10 | 642 ± 9 | 560 ± 8 | 654 ± 8 | 558 ± 11 | 639 ± 9 | 543 ± 6 | 628 ± 10 | 0.965 |

All data are represented as mean ± SEM, n=12/group. *: p value of the ANOVA realized on changes between the beginning and the end of the experimental period. a,b Mean values with unlike letters were significantly different (p < 0.05).

**Table S6.** **Plasma and liver levels of major polyunsaturated fatty acids (PUFA)**. LDLR^-/-^ mice were given by daily oral gavages (20 weeks) either oleic acid rich sunflower oil (Control group) or a mixture of oleic acid rich sunflower oil and DHA rich tuna oil providing 0.1%, 1% or 2% of energy as DHA (DHA1, DHA2, and DHA3 groups respectively).

|  | **Control** | **DHA1** | **DHA2** | **DHA3** | **P value** |
| --- | --- | --- | --- | --- | --- |
| **Plasma PUFA (% total FA)** |  |  |  |  |  |
| 18:2 n-6 | 10.79 ± 0.18 ^a^ | 11.22 ± 0.36 ^ac^ | 12.99 ± 0.30 ^bc^ | 12.44 ± 0.16 ^c^ | <0.001 |
| 18:3 n-3 | 0.16 ± 0.01 ^a^ | 0.22 ± 0.01 ^b^ | 0.25 ± 0.01 ^bc^ | 0.26 ± 0.01 ^c^ | <0.001 |
| 20:4 n-6 (AA) | 15.84 ± 0.68 ^a^ | 10.35 ± 0.62 ^ab^ | 5.05 ± 0.23 ^bc^ | 4.21 ± 0.11 ^c^ | <0.001 |
| 20:5 n-3 (EPA) | 0.30 ± 0.02 ^a^ | 0.86 ± 0.08 ^ab^ | 5.08 ± 0.40 ^bc^ | 7.03 ± 0.36 ^c^ | <0.001 |
| 22:6 n-3 (DHA) | 3.57 ± 0.10 ^a^ | 5.35 ± 0.30 ^ab^ | 9.28 ± 0.51 ^bc^ | 11.56 ± 0.75 ^c^ | <0.001 |
|  |  |  |  |  |  |
| **Liver PUFA(% total FA)** |  |  |  |  |  |
| 18:2 n-6 | 7.29 ± 0.42 ^a^ | 8.28 ± 0.41 ^ab^ | 9.52 ± 0.50 ^bc^ | 10.39 ± 0.42 ^c^ | <0.001 |
| 18:3 n-3 | 0.33 ± 0.03 | 0.39 ± 0.04 | 0.38 ± 0.04 | 0.37 ± 0.05 | ns |
| 20:4 n-6 (AA) | 3.09 ± 0.37 | 3.19 ± 0.71 | 1.98 ± 0.19 | 2.37 ± 0.28 | ns |
| 20:5 n-3 (EPA) | 0.06 ± 0.01 ^a^ | 0.27 ± 0.05 ^ab^ | 1.83 ± 0.24 ^bc^ | 2.94 ± 0.18 ^c^ | <0.001 |
| 22:6 n-3 (DHA) | 1.06 ± 0.17 ^a^ | 2.23 ± 0.57 ^ab^ | 5.37 ± 0.82 ^bc^ | 8.97 ± 1.01 ^c^ | <0.001 |

All data are represented as mean ± SEM, n=10/group. a,b,c Mean values with unlike letters were significantly different (p < 0.05).

**Table S7**. **Plasma levels of PUFAs-derived oxylipins.** LDLR^-/-^ mice were given by daily oral gavages (20 weeks) either oleic acid rich sunflower oil (Control group) or a mixture of oleic acid rich sunflower oil and DHA rich tuna oil providing 0.1%, 1% or 2% of energy as DHA (DHA1, DHA2, and DHA3 groups respectively).

|  | **Control** | **DHA1** | **DHA2** | **DHA3** | **P value** |
| --- | --- | --- | --- | --- | --- |
| **Plasma oxylipins (nM)** |  |  |  |  |  |
| 20:4 n-6 Epoxides | 1761.20 ± 280.00 ^a^ | 2230.11 ± 494.61 ^ab^ | 762.87 ± 124.97 ^bc^ | 694.79 ± 105.79 ^c^ | <0.001 |
| 14(15)-EpETrE | 783.00 ± 150.26 | 1123.97 ± 278.67 | 376.41 ± 69.31 | 372.99 ± 69.43 | 0.018 |
| 11(12)-EpETrE | 751.40 ± 107.89 ^a^ | 860.78 ± 172.11 ^a^ | 302.14 ± 46.04 ^b^ | 245.00 ± 30.74 ^b^ | <0.001 |
| 8(9)-EpETrE | 226.80 ± 24.46 ^a^ | 245.67 ± 46.09 ^a^ | 84.32 ± 11.69 ^b^ | 76.80 ± 7.98 ^b^ | <0.001 |
| 20:4 n-6 Diols | 69.17 ± 3.48 ^a^ | 62.05 ± 3.32 ^a^ | 29.33 ± 4.70 ^b^ | 23.16 ± 1.89 ^b^ | <0.001 |
| 14,15-DiHETrE | 1.62 ± 0.09 ^a^ | 1.05 ± 0.07 ^ab^ | 0.55 ± 0.04 ^bc^ | 0.52 ± 0.03 ^c^ | <0.001 |
| 11,12- DiHETrE | 1.93 ± 0.10 ^a^ | 1.30 ± 0.11 ^ab^ | 0.70 ± 0.06 ^bc^ | 0.56 ± 0.04 ^c^ | <0.001 |
| 8,9- DiHETrE | 6.69 ± 0.44 ^a^ | 5.22 ± 0.47 ^ab^ | 2.66 ± 0.22 ^bc^ | 1.79 ± 0.14 ^c^ | <0.001 |
| 5,6-DiHETrE | 58.93 ± 3.55 ^a^ | 54.48 ± 2.82 ^ab^ | 25.43 ± 4.43 ^bc^ | 20.29 ± 1.75 ^c^ | <0.001 |
| 20:4 n-6 Alcohols | 543.15 ± 72.52 ^a^ | 457.30 ± 78.81 ^a^ | 185.34 ± 17.30 ^b^ | 165.04 ± 19.12 ^b^ | <0.001 |
|  |  |  |  |  |  |
| 20-HETE | 56.84 ± 4.23 ^a^ | 39.58 ± 4.51 ^a^ | 18.06 ± 2.79 ^b^ | 12.29 ± 1.00 ^b^ | <0.001 |
| 15- HETE | 57.60 ± 7.65 ^a^ | 42.18 ± 5.51 ^a^ | 14.73 ± 2.24 ^b^ | 10.33 ± 2.43 ^b^ | <0.001 |
| 12- HETE | 139.58 ± 42.36 | 162.04 ± 55.60 | 51.73 ± 8.81 | 66.94 ± 15.01 | 0.208 |
| 11- HETE | 40.20 ± 4.21 ^a^ | 30.67 ± 3.58 ^a^ | 14.04 ± 1.30 ^b^ | 10.98 ± 1.77 ^b^ | <0.001 |
| 8-HETE | 51.63 ± 4.25 ^a^ | 38.58 ± 3.54 ^ab^ | 16.53 ± 2.18 ^bc^ | 10.80 ± 1.53 ^c^ | <0.001 |
| 9- HETE | 41.40 ± 4.71 ^a^ | 31.11 ± 4.25 ^ab^ | 17.43 ± 1.13 ^bc^ | 15.22 ± 1.84 ^c^ | <0.001 |
| 5- HETE | 155.90 ± 13.91 ^a^ | 113.14 ± 10.37 ^ab^ | 52.82 ± 5.21 ^bc^ | 38.48 ± 3.86 ^c^ | <0.001 |
| 20:5 n-3 Epoxides | 44.15 ± 8.81 ^a^ | 253.83 ± 67.56 ^ab^ | 782.00 ± 112.46 ^bc^ | 1149.90 ± 187.19 ^c^ | <0.001 |
| 17(18)-EpETE | 25.99 ± 5.47 ^a^ | 144.88 ± 36.74 ^ab^ | 466.40 ± 65.88 ^bc^ | 677.00 ± 107.74 ^c^ | <0.001 |
| 14(15)-EpETE | 18.15 ± 3.39 ^a^ | 108.96 ± 30.90 ^ab^ | 315.60 ± 47.09 ^bc^ | 472.90 ± 80.04 ^c^ | <0.001 |
| 20:5 n-3 Diols | 4.09 ± 0.37 ^a^ | 5.56 ± 0.49 ^ab^ | 17.06 ± 0.86 ^bc^ | 22.76 ± 1.55 ^c^ | <0.001 |
| 17,18-DiHETE | 2.68 ± 0.24 ^a^ | 3.78 ± 0.46 ^ab^ | 12.38 ± 0.65 ^bc^ | 17.51 ± 1.29 ^c^ | <0.001 |
| 14,15-DiHETE | 1.42 ± 0.16 ^a^ | 1.78 ± 0.18 ^a^ | 4.68 ± 0.32 ^b^ | 5.25 ± 0.39 ^b^ | <0.001 |
| 20:5 n-3 Alcohols | 11.25 ± 1.87 ^a^ | 26.50 ± 5.13 ^ab^ | 118.15 ± 13.40 ^bc^ | 188.01 ± 17.28 ^c^ | <0.001 |
|  |  |  |  |  |  |
| 15-HEPE | 1.10 ± 0.20 ^a^ | 2.88 ± 0.57 ^a^ | 11.63 ± 1.52 ^b^ | 13.43 ± 1.37 ^b^ | <0.001 |
| 12-HEPE | 6.07 ± 1.51 ^a^ | 15.75 ± 3.83 ^ab^ | 66.61 ± 10.98 ^bc^ | 136.27 ± 17.76 ^c^ | <0.001 |
| 5-HEPE | 4.08 ± 0.62 ^a^ | 7.86 ± 1.14 ^a^ | 39.91 ± 4.82 ^b^ | 38.31 ± 4.55 ^b^ | <0.001 |
| 22:6 n-3 Epoxides | 904.94 ± 129.08 ^a^ | 2129.44 ± 433.65 ^ab^ | 2030.90 ± 264.35 ^ab^ | 2796.80 ± 363.25 ^b^ | 0.002 |
| 19(20)-EpDPE | 678.70 ± 100.07 ^a^ | 1564.44 ± 293.96 ^a^ | 1452.30 ± 199.17 ^a^ | 2041.90 ± 240.45 ^b^ | 0.002 |
| 16(17)-EpDPE | 226.24 ± 31.23 ^a^ | 565.00 ± 140.51 ^a^ | 578.60 ± 75.60 ^b^ | 754.90 ± 125.58 ^b^ | 0.004 |
| 22:6 n-3 Diol (19,20-DiHDPA) | 5.77 ± 0.33 ^a^ | 9.24 ± 0.72 ^ab^ | 13.73 ± 0.66 ^bc^ | 16.85 ± 0.96 ^c^ | <0.001 |
| 22:6 n-3 Alcohol (17-HDoHE) | 21.48 ± 2.74 ^a^ | 37.80 ± 5.46 ^a^ | 58.91 ± 5.17 ^b^ | 69.55 ± 6.45 ^b^ | <0.001 |
| 20:3 n-6-Derived oxylipins | 5.96 ± 2 ^a^ | 6.73 ± 1.5 ^a^ | 4.2 ± 1.6 ^ab^ | 3.59 ± 1.6 ^b^ | <0.001 |
| 18:2 n-6-Derived oxylipins |  |  |  |  |  |
| 12(13)-EpOME | 283 ± 110 | 453 ± 260 | 378 ± 140 | 321 ± 110 | ns |
| 9(10)-EpOME | 285 ± 110 | 435 ± 230 | 373 ± 150 | 301 ± 97 | ns |
| 12,13-DiHOME | 4.11 ± 0.77 | 3.94 ± 1.1 | 3.43 ± 0.93 | 3.71 ± 0.49 | ns |
| 9,10-DiHOME | 33.4 ± 5.7 ^a^ | 30.6 ± 7.2 ^a^ | 23.6 ± 4.3 ^b^ | 21.8 ± 4.5 ^b^ | <0.001 |
| 13-HODE | 344 ± 98 | 400 ± 140 | 337 ± 73 | 324 ± 110 | ns |
| 9-HODE | 71.4 ± 19 | 73.6 ± 21 | 72.1 ± 13 | 62.5 ± 24 | ns |
| 18:3 n-3-Derived oxylipins |  |  |  |  |  |
| 15(16)-EpODE | 30.4 ± 11 | 54.6 ± 30 | 44.8 ± 20 | 37.3 ± 16 | ns |
| 12(13)-EpODE | 3.04 ± 0.95 | 6.73 ± 4 | 5.62 ± 2.6 | 5.47 ± 1.9 | <0.05 |
| 9(10)-EpODE | 21.7 ± 11 | 41.6 ± 23 | 33.6 ± 13 | 28.6 ± 13 | ns |
| 9-HOTE | 2.05 ± 0.69 | 2.27 ± 0.82 | 2.2 ± 0.57 | 1.89 ± 0.51 | ns |
| 13-HOTE | 3.78 ± 1.6 | 3.61 ± 1.3 | 3.6 ± 1 | 4.06 ± 1 | ns |

All data are represented as mean ± SEM, n=10/group. a,b,c Mean values with unlike letters were significantly different (p < 0.05).

**Table S8: Thromboxane and prostaglandin stability through sample processing** *^a^*

|  | **Hydrolysis + SPE** | **SPE** | **Standards** | **SSTD** |
| --- | --- | --- | --- | --- |
| TXB2 *^b^* | 24.5 ± 6.4 % | 101 ± 1.5 % | 101 ± 0.77 % | d4-TXB2 |
| 6-keto PGF1a | 101 ± 0.92 % | 102 ± 1.2 % | 102 ± 0.61 % | d4-6-keto PGF1a |
| PGE1 *^b^* | ND | 106 ± 3.6 % | 101 ± 0.53 % | d4-PGD2 |
| PGE2 *^b^* | ND | 103 ± 1.6 % | 99.5 ± 2 % | “” |
| PGE3 *^b^* | ND | 100 ± 2.9 % | 101 ± 0.92 % | “” |
| PGD2 *^b^* | ND | 101 ± 1.6 % | 99.2 ± 0.31 % | “” |
| 15-deoxy PGJ2 *^b^* | ND | 115 ± 6.2 % | 96.1 ± 0.4 % | “” |
| PGJ2/ δ 12-PGJ2 *^b^* | ND | 96.1 ± 2.7 % | 98 ± 0.5 % | “” |
| PGF2a / F2-IsoP | 108 ± 0.81 % | 105 ± 1.1 % | 105 ± 1.1 % | d4-PGF2a |

*a* - A high level calibration standard was subjected to solid phase extraction with or without prior exposure to base hydrolysis procedures and compared to triplicate analyses of calibration standards. Results are means ± SD relative to theoretical concentrations with adjustment for surrogate recoveries.

*b* - As previously reported, the thromboxanes and beta-hydroxy-keto prostanoids are unstable under alkali conditions.

**Table S9: Fatty acid triol stability through sample processing** *^a^*

|  | **Hydrolysis + SPE** | **SPE** | **Standards** | **SSTD** |
| --- | --- | --- | --- | --- |
| 20-hydroxy-LTB4 | 95.4 ± 1.3 % | 93.8 ± 1.5 % | 100 ± 0.93 % | d4 6-keto PGF1a |
| 9,12,13-TriHOME | 116 ± 3.5 % | 112 ± 3.9 % | 98.7 ± 1.1 % | d4-6-keto PGF1a |
| 9,10,13-TriHOME | 122 ± 1.7 % | 119 ± 8 % | 102 ± 2.7 % | d4-6-keto PGF1a |
| 11,12,15-THET | 106 ± 2.5 % | 92.6 ± 1.4 % | 101 ± 1.3 % | d4-PGF2a |
| Resolvin E1 | 92.1 ± 2.4 % | 90.7 ± 0.28 % | 101 ± 0.58 % | d4-6-keto PGF1a |
| Resolvin D1 *^b^* | 74.3 ± 7.4 % | 52.5 ± 1.8 % | 103 ± 1.2 % | d4-PGF2a |
| Lipoxin A4 *^b^* | 78.8 ± 2.9 % | 61.5 ± 2 % | 104 ± 1.3 % | d4-PGF2a |

*a* - A high level calibration standard was subjected to solid phase extraction with or without prior exposure to base hydrolysis procedures and compared to triplicate analyses of calibration standards. Results are means ± SD relative to theoretical concentrations with adjustment for surrogate recoveries.

*b* - Poor analyte recoveries suggest a reduced accuracy in results for these analytes using the current surrogate spiking/tagging regimen. However, high precision suggests that treatment effects within the study cohort should be robustly detectable.

**Table S10: Fatty acid diol stability through sample processing** *^a^*

|  | **Hydrolysis + SPE** | **SPE** | **Standards** | **SSTD** |
| --- | --- | --- | --- | --- |
| 20-carboxy-LTB4 | 82.2 ± 1.6 % | 73.3 ± 1.4 % | 97.4 ± 1.3 % | d4 6-keto PGF1a |
| LTB4 | 92.8 ± 6.4 % | 97.7 ± 13 % | 92.2 ± 4.3 % | d4-LTB4 |
| 6-trans-LTB4 | 88.3 ± 8.1 % | 88.5 ± 9.9 % | 94 ± 6.8 % | d4-LTB4 |
| LTB5 | 94.1 ± 6.9 % | 103 ± 14 % | 94.1 ± 5.7 % | d4-PGF2a |
| 8,15-DiHETE | 99.6 ± 5.5 % | 102 ± 4.9 % | 95.4 ± 2.1 % | d11-14,15-DiHETrE |
| 5,15-DiHETE *^b^* | 37.7 ± 1.7 % | 107 ± 1.7 % | 95.8 ± 0.33 % | d11-14,15-DiHETrE |
| 12,13-DiHOME | 112 ± 12 % | 135 ± 7.5 % | 93.4 ± 0.53 % | d11-14,15-DiHETrE |
| 9,10-DiHOME | 117 ± 10 % | 134 ± 4.7 % | 96.6 ± 1.2 % | d11-14,15-DiHETrE |
| 15,16-DiHODE | 116 ± 9.6 % | 133 ± 5.2 % | 100 ± 0.1 % | d11-14,15-DiHETrE |
| 12,13-DiHODE | 111 ± 9.7 % | 131 ± 4.4 % | 96.2 ± 0.56 % | d11-14,15-DiHETrE |
| 9,10-DiHODE | 111 ± 11 % | 132 ± 5.1 % | 96.8 ± 0.92 % | d11-14,15-DiHETrE |
| 14,15-DiHETrE | 95.1 ± 2.4 % | 96.3 ± 0.92 % | 94.9 ± 1.1 % | d11-14,15-DiHETrE |
| 11,12-DiHETrE | 93.3 ± 2.9 % | 93.1 ± 0.92 % | 97.9 ± 2.7 % | d11-14,15-DiHETrE |
| 8,9-DiHETrE | 103 ± 8.4 % | 99.4 ± 0.31 % | 110 ± 2.7 % | d11-14,15-DiHETrE |
| 5,6-DiHETrE | 127 ± 2.3 % | 78.4 ± 1.3 % | 98.1 ± 0.31 % | d11-14,15-DiHETrE |
| 17,18-DiHETE | 122 ± 1.6 % | 111 ± 4.5 % | 99 ± 5.4 % | d11-14,15-DiHETrE |
| 14,15-DiHETE | 119 ± 5.5 % | 108 ± 1.7 % | 105 ± 2.3 % | d11-14,15-DiHETrE |
| 19,20-DiHDoPA | 83.2 ± 4.3 % | 72.2 ± 2.7 % | 100 ± 0.23 % | d11-14,15-DiHETrE |

*a* - A high level calibration standard was subjected to solid phase extraction with or without prior exposure to base hydrolysis procedures and compared to triplicate analyses of calibration standards. Results are means ± SD relative to theoretical concentrations with adjustment for surrogate recoveries.

*b* - Poor analyte recoveries suggest a reduced accuracy in results for these analytes using the current surrogate spiking/tagging regimen. However, high precision suggests that treatment effects within the study cohort should be robustly detectable.

**Figure S1. Correlations between doses of DHA given to LDLR^-/-^ mice and plasma levels of PUFA and of AA, EPA, and DHA and their corresponding oxylipins.**

**Figure S2. Correlations between plasma levels of EPA, and their corresponding specific oxylipins.**

**Figure S3. Correlations between plasma levels of DHA, and their corresponding specific oxylipins.**

**Figure S4. Correlations between doses of DHA given to LDLR^-/-^ mice and liver levels of PUFA and plasma levels of n-6 PUFA, n-3 PUFA and DHA and their corresponding peroxidized metabolites.**

**Figure S5. Partial least squares discrimination analysis of dietary groups in LDLR^-/-^ given by daily oral gavages increasing doses of DHA. A**) Animals eating each dietary mixture with complete data sets (n=10/group) were segregated by PLS analyses. **B**) Group segregation was driven by plasma (yellow triangle) and liver (orange triangle) metabolites belonging to clusters C2, C3, and C4 as identified in Figure 7. The plasma EPA metabolite, 17,18-dihydroxyeicosaotetraenoic acid (17,18-DiHETE) and the liver DHA metabolite group F4-neuroprostanes (F4-NeuroPs) were the most frequently selected variables occurring in 80% and 60% of models, respectively.


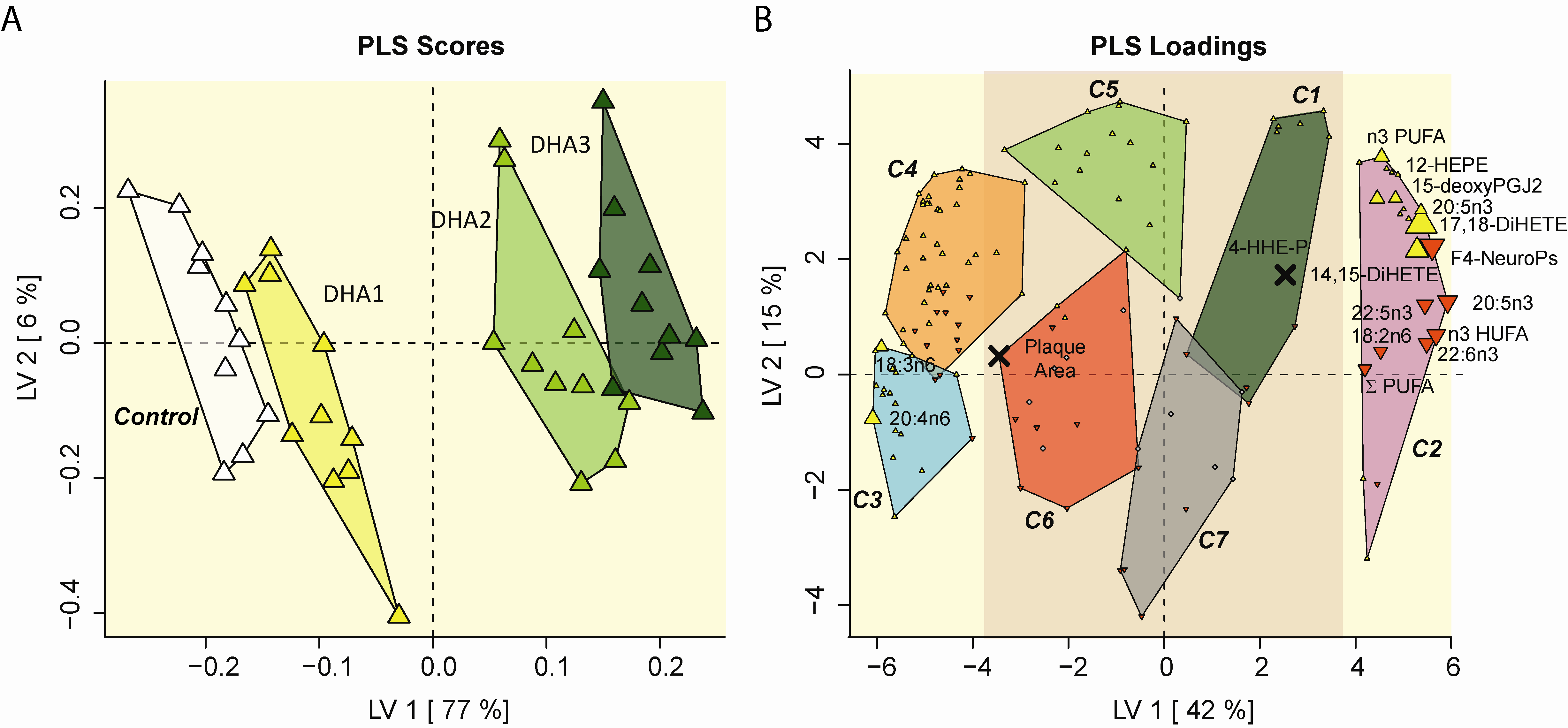


**Figure S6. Correlation between plasma F2-isoprostanes and 9-HETE levels.**

**Figure S7. Correlations between plasma oxylipins and plasma 9-HETE levels.**
